# Interaction of HIV-1 integrase with polypyrimidine tract binding protein and associated splicing factor (PSF) and its impact on HIV-1 replication

**DOI:** 10.1186/s12977-019-0474-1

**Published:** 2019-04-29

**Authors:** Pooja Yadav, Souvik Sur, Dipen Desai, Smita Kulkarni, Vartika Sharma, Vibha Tandon

**Affiliations:** 1 Department of Chemistry, University of Delhi, Delhi, 110007 India; 2 Special Center for Molecular Medicine, Jawaharlal Nehru University, New Delhi, 110067 India; 3 National AIDS Research Institute, Pune, Maharashtra 411026 India; 4 International Centre for Genetics Engineering and Biotechnology, New Delhi, 110067 India

**Keywords:** PSF, HIV, HIV-1 integrase, Host–pathogen interaction

## Abstract

**Background:**

The different interactions between viral proteins and cellular host proteins are required for efficient replication of HIV-1. Various reports implicated host cellular proteins as a key factor that either interact directly with HIV-1 integrase (IN) or get involved in the integration process of virus resulting in the modulation of integration step. Polypyrimidine tract binding protein and associated splicing factor (PSF) has diverse functions inside the cell such as transcriptional regulation, DNA repair, acts as nucleic acids binding protein and regulate replication and infectivity of different viruses.

**Results:**

The protein binding study identified the association of host protein PSF with HIV-1 integrase. The siRNA knockdown (KD) of PSF resulted in increased viral replication in TZM-bl cells, suggesting PSF has negative influence on viral replication. The quantitative PCR of virus infected PSF knockdown TZM-bl cells showed more integrated DNA and viral cDNA as compared to control cells. We did not observe any significant difference between the amount of early reverse transcription products as well as infectivity of virus in the PSF KD and control TZM-bl cells. Molecular docking study supported the argument that PSF hinders the binding of viral DNA with IN.

**Conclusion:**

In an attempt to study the host interacting protein of IN, we have identified a new interacting host protein PSF which is a splicing factor and elucidated its role in integration and viral replication. Experimental as well as in silico analysis inferred that the host protein causes not only change in the integration events but also targets the incoming viral DNA or the integrase-viral DNA complex. The role of PSF was also investigated at early reverse transcript production as well as late stages. The PSF is causing changes in integration events, but it does not over all make any changes in the virus infectivity. MD trajectory analyses provided a strong clue of destabilization of Integrase-viral DNA complex occurred due to PSF interaction with the conserved bases of viral DNA ends that are extremely crucial contact points with integrase and indispensable for integration. Thus our study emphasizes the negative influence of PSF on HIV-1 replication.

**Electronic supplementary material:**

The online version of this article (10.1186/s12977-019-0474-1) contains supplementary material, which is available to authorized users.

## Background

The HIV-1 life cycle includes two essential processes, reverse transcription, forming the linear double stranded DNA (cDNA) and the integration of viral dsDNA into host genome. The integration process is catalyzed by viral protein integrase which utilizes different host proteins for integration of viral DNA [1]. Identification of these interacting host cellular protein provide better understanding of mechanism of viral replication and subsequently development of new therapeutic approaches. Host factors such as Integrase interactor-1 [2, 3], High Mobility Group Protein (HMGA-1) [4], Barrier to Autointegration (BAF) [5], Lens epithelium derived growth factor (LEDGF) [6] were observed to interact with HIV-1 integrase (IN) and aid in it’s activity. It is reported that LEDGF also interact with splicing factors and enhance the integration process [7]. Importin α [8–10], Transportin 3 protein (TNPO3) [11,12] have been reported in nuclear import of Preintegration complex (PICs) of HIV-1, while Dynein light chain 1 (DYNLL1) [13], LEDGF, Polycomb protein EED [14] Rad 18 [15] all interacts and co-localizes with IN inside the nucleus. The DNA repair mechanism such as Homologous recombination (HR), Non-homologous end joining (NHEJ) and Base excision repair pathway has major impact on integration frequency. They are involved in DNA repair during retroviral integration process as well as viral Long terminal repeat (2-LTR) formation, which occurs in the absence of viral DNA integration.

In the present study, we have identified a new HIV-1 integrase interacting protein, polypyrimidine tract binding protein and associated splicing factor (PSF) through pull down assay and co immunoprecipitation of IN with mammalian cell protein. Splicing is major event in the propagation of HIV-1. Splicing factor such as Serine Arginine rich splicing factor (SRSF1) [16] regulate HIV-1 transcription whereas, Small nuclear RNAs [17] inhibit HIV-1 replication through excessive RNA Splicing. RNA helicases has dual mode of action, one way it triggers antiviral response in host and other way it promotes viral gene expression also [18]. PSF is a multifunctional protein. Apart from its role in splicing process [19, 20], it along with p54nrb/NonO plays role in transcriptional regulation [21], and acts as bridge molecule between nuclear proteins and RNA polymerase II [22]. At the same time PSF: p54 complex is involved in transcriptional repression also. PSF promotes viral RNA transcript production through interaction with viral RNA and MATR3 [23]. The other important roles of PSF are nucleic acid binding protein [24, 25], interaction with RAD51 to facilitate repair of double strand breaks [26]. PSF is one of the key factors mediating the posttranscriptional regulation of HIV-1. PSF causes a dose-dependent inhibition of virus production in cell culture, pointing to the possibility that PSF can contribute to the control of HIV-1 propagation in vivo [27]. It also forms paraspeckles that are crucial in controlling gene expression in multiple cellular processes especially during viral infection, stress or differentiation.

We studied PSF interaction with IN, because of its high score in mass spectrometry through His pull down assay. In an attempt to reveal how PSF participate in integration of viral DNA in host DNA and its replication, we first examined it’s impact on HIV-1 replication and demonstrated that siRNA mediated PSF knockdown significantly increases viral replication in TZM-bl cells. However, qPCR data analysis revealed its association with viral DNA also. Our studies suggest that PSF negatively regulates the viral replication in TZM-bl cells. The viral 2-LTR, integrated provirus increased in the PSF knockdown TZM-bl cells. The quantity of HIV-1 cDNA also got increased in PSF knockdown cells and vice versa. PSF however was not found to affect post-integration steps in viral replication cycle. In order to reveal how PSF affects the integration events, we performed molecular docking and simulation studies with ternary complex of PSF: IN: viral dsDNA and binary complex i.e.; PSF:IN. We hereby hypothesize that IN, PSF and viral DNA form a ternary complex during integration process to cross talk with each other.

## Results

### Isolation and identification of cellular protein interacting with purified IN

**Fig. 1:**
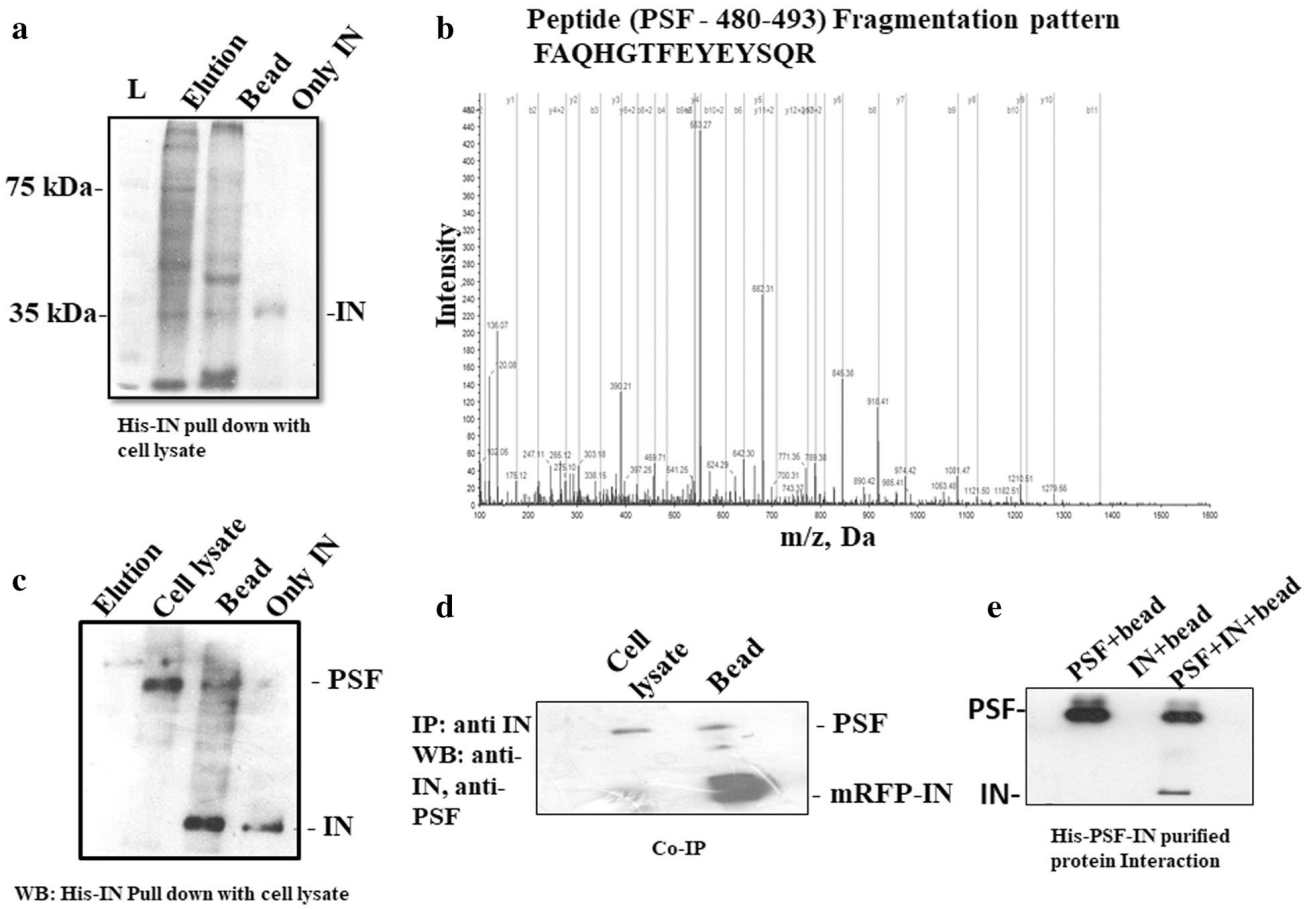
**a** Interaction between host cellular proteins and HIV-1 IN was detected by pull down assay. Elution lane consists of protein obtained during washing. Interacted proteins bound to the bead was shown in lane bead **b** Cellular SFPQ/PSF fragmentation pattern of peptide FAQHGTFEYEYSQR. The ‘b” and “y” ion series derived from the amide bond cleavage during collision induced dissociation of the peptide provide amino acid sequence information. **c** The interaction between IN and PSF was confirmed by western blot after performing pull down assay of IN with HeLa cell nuclear protein. The proteins were detected with anti-IN and anti-PSF antibody. **d** Co-immunoprecipitation of mRFP-tagged IN with PSF/SFPQ from nuclear extracts: mRFP-integrase was transfected in HeLa cell line. The cell lysate was then incubated with anti-IN antibody and protein A agarose beads for 4–5 h at 4 °C. The eluted fractions were detected by PSF and IN antibody. Lane bead consists of interacting proteins bound to the bead. Cell lysate consists of only cellular protein with Ni–NTA beads before transfection. **e** Purified protein–protein interaction detected by western blot. The lanes consists of beads bound to the purified protein was checked for interaction with both IN and PSF in Lanes: PSF + bead—consists of PSF bound to Ni–NTA bead, lane IN + bead—Unbound IN protein to Ni–NTA bead. IN + PSF + bead lane is His-PSF and IN interaction obtained after washing in a Ni^+2^-NTA affinity bead

The pull down assay of purified Histidine tagged IN (His-IN) protein with HeLa cell proteins were performed to identify the interacting proteins and the unbound proteins obtained during wash step (Fig. 1a). Several previously reported proteins known to interact and influence the activity of IN proteins such as LEDGF and importin 5 were identified by us through MALDI and LC/MS/MS (Table 1). Along with these IN interacting protein, other proteins identified by mass spectrometry were Matrin 3 (MATR3), p54nrb/NonO, polypyrimidine tract, DNA Topoisomerase 1 (Table 1). These proteins are the interacting partner of PSF and probably the reason for its identification in the pull down assay. MATR3 associates with PSF and acts as a cofactor in viral RNA export mediated by rev protein. DNA topoisomerase 1 interacts with PSF and implied in RNA splicing as well as stimulation of splicing factor by phosphorylation. Its activity has been found to enhanced to fivefold when it is complexed with PSF [28]. The p54nrb is highly homologous to the PSF C-terminal region. However, we selected PSF and not p54nrb as we found PSF as a new interacting protein which is already reported in HIV-1 downregulation [27] but whose detailed functional analysis has not been understood. Heterogenous nuclear ribonuclear protein (hnRPK) has been identified to interact with LEDGF and involved in mRNA processing along with other splicing factors [29].

**Table 1:** Identified integrase interacting protein by MS/MS analysis

Sl. no.	Sequence	Identified Protein	Function	Prot score
1.	FAQHGTFEYEYSQR	PSF	Involved in splicing	12
2.	VDEVPDGAVKPPTNK	LEDGF	Transcriptional coactivator	2
3.	SLVEIADTVPK	Importin-5	Import of protein inside the nucleus	2
4.	TDYNASVSVPDSSGPER	HNRP K protein	In premRNA processing	4
5.	GPVFAPPYEPLPENV	DNA Topoisomerase 1	Involved in DNA unwinding, supercoiling and replication	6.25
6.	GPSLNPVLDYDHGSR	Matrin-3	Interacts with nuclear matrix protein and forms fibrogranular network	2
7.	AQPGSFEYEYAMR	NONO	Involved in pre mRNA splicing, transcription process	14
8.	LPSGDSQPSLDQTMAAAFG	Polypyrimidine tract binding protein-1	In pre mRNA splicing	17.3

PSF had a prot score of 12 and LC/MS/MS identified peptides which matched with PSF covering 18.9 of the sequence (Table 2). The fragmentation pattern of a peptide of PSF is depicted in Fig. 1b. The IN–PSF protein interaction was again confirmed by western blot (Fig. 1c) and the cell based co-immunoprecipitation (Co-IP) assay (Fig. 1d). Thus, pull down assay and LC/MS/MS suggested the interaction of PSF with IN. The interaction study using purified protein by Ni^+2^–NTA affinity chromatography provided direct evidence for the interaction between both the proteins (Fig. 1e and Additional file 1: Fig S1). To analyze the association further between both the proteins, co-transfection of both the plasmid was carried out.

**Table 2:** In-gel digests of PSF tryptic peptide, confirmed by MS/MS Analysis

Residue	Sequence	Theoritical MW	Observed MW
301–314	LFVGNLPADITEDEFK	1806.9039	1806.9106
320–330	YGEPGEVFINK	1251.6135	1251.6177
382–399	VSNELLEEAFSQFGPIER	2064.0164	2064.0256
331–362	PVIVEPLEQLDDEDGLPEK	2134.0681	2134.0745
480–493	FAQHGTFEYEYSQR	1761.7747	1761.7833

*MW* molecular weight

### PSF colocalizes with IN

**Fig. 2:**
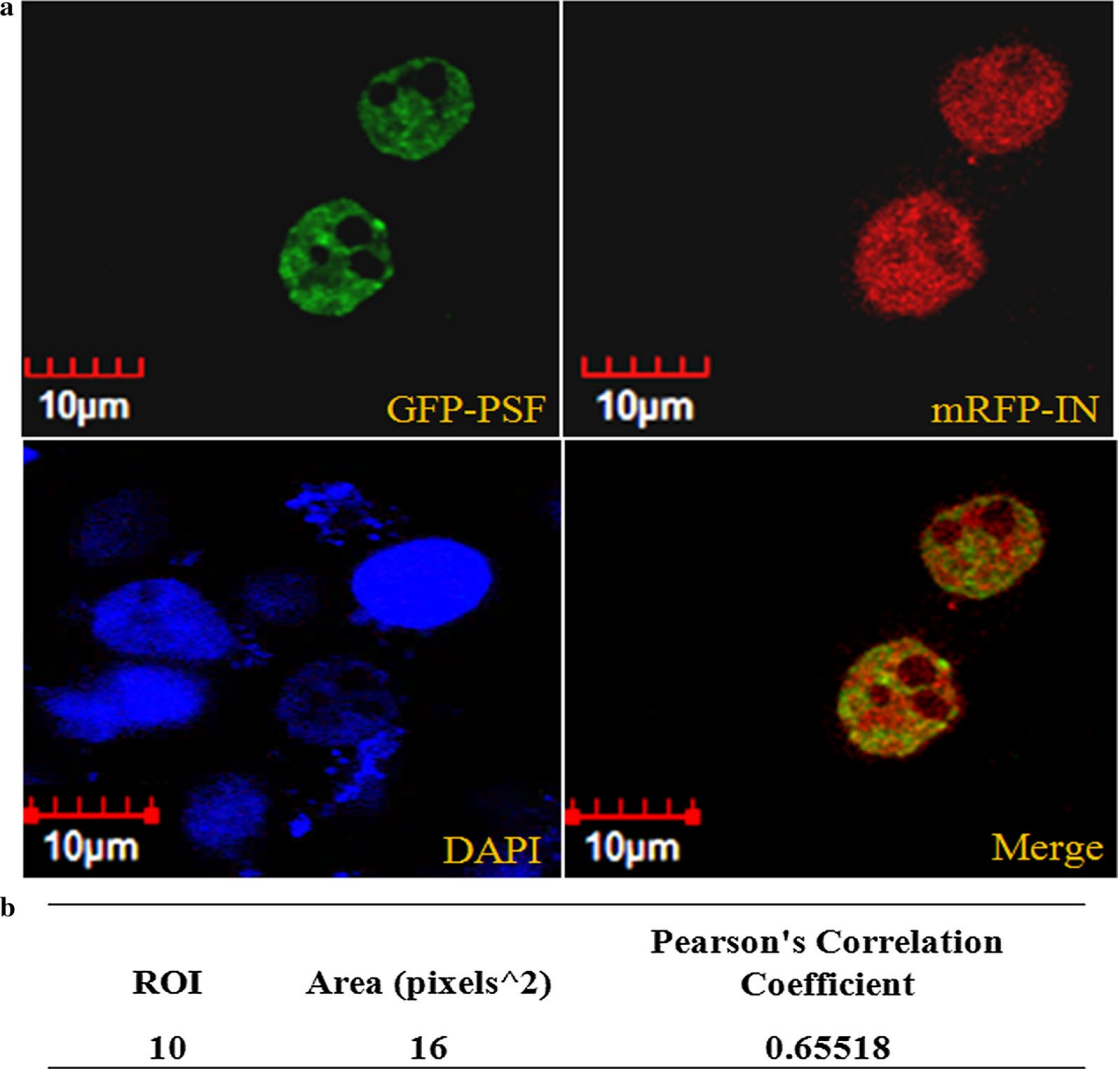
Confocal Microscopy of subcellular compartments for classical colocalization of GFP-PSF and mRFP-IN was performed in HeLa cells. Significant colocalization of both the proteins was observed after 24 h which was analysed through pearson correlation coefficient (shown in supporting). DAPI was used to stain the nuclear DNA

The subcellular distribution pattern of GFP tagged PSF and mRFP tagged IN was studied by fluorescence microscopy. The GFP tagged PSF localization was observed to be completely nuclear however mRFP tagged IN was observed in both nucleus and cytoplasm (Fig. 2a). The microscopic fluorescence image revealed the significant intranuclear co-localization of both protein at 24 and 48 h in both the cell line (Fig. 2a). Pearson correlation coefficient (PCC) was used to measure linear correlation or degree of colocalization between two different variables for e.g. between two fluorophores. Ten region of interest (ROI) from the merged image of cotransfected cells were selected. The region of interest (ROI) were examined for different fluorophores in the same pixel using two different channels. The average pearson coefficient was observed to be 0.655 which suggests significant positive linear correlation between the two fluorophores (Fig. 2b).

### In vitro IN activity is not obstructed by PSF

**Fig. 3:**
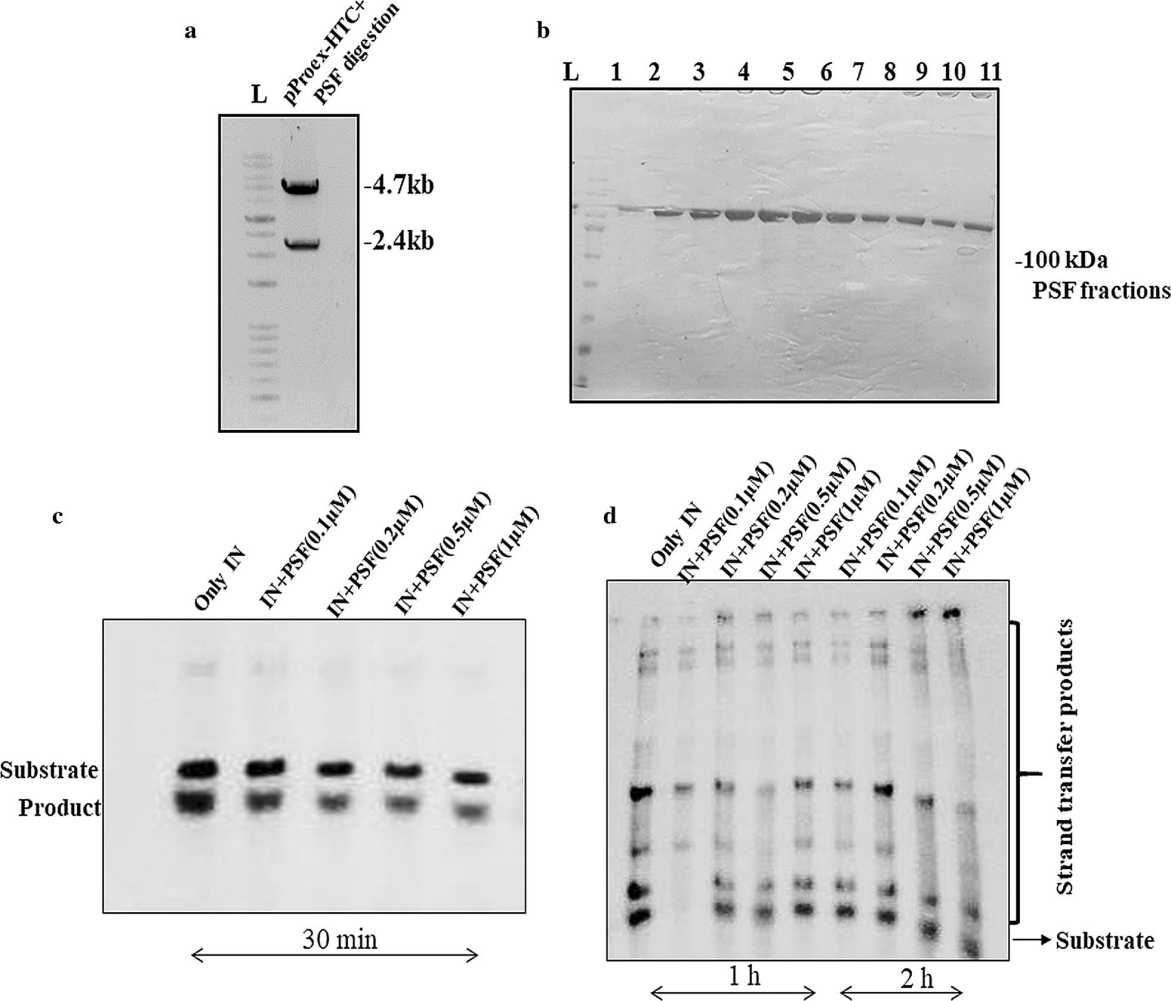
Cloning of PSF in bacterial expression system vector and in vitro IN activity assay. **a** The amplified PSF PCR product was digested with Kpn1 and Sac1 restriction enzyme and ligated with pPROEX-HTC bacterial expression vector. **b** Purified fractions of PSF by Nickel-NTA affinity chromatography. **c** 3′ processing assay was performed using IN (250 nM) and PSF and 0.5 pmol of radiolabeled oligos. PSF concentration varied from 0.1, 0.2, 0.5, 1 µM in the lanes and incubated for 30 min. Lane 1 contains only IN and oligos. **d** Strand transfer activity was done using oligos mentioned in method section. The lanes contains IN (250 nM) and PSF from 0.1 to 1 µM but incubated at 1 and 2 h respectively. Lane 1 contains only IN (250 nM) and oligos. The reaction products were analyzed by electrophoresis on 15% polyacrylamide gels with 8 M urea in Tris borate—EDTA, pH 7.6, and autoradiographed

The in vitro 3′processing (3′P) and strand transfer activity (STA) assay of IN was performed by autoradiography using 0.5 pmol of oligos labeled at 5′end with [γ-^32^P] ATP with the help of polynucleotide kinase. The entire gene of PSF was cloned in bacterial expression vector pPROEX-HTc (Fig. 3a) and purified by Ni–NTA affinity chromatography (Fig. 3b). The His-IN was also purified by Nickel–Nitrilotriacetic acid (Ni–NTA) affinity column chromatography. We have used different concentration of purified PSF protein to analyse its influence on the 3′P and STA assay of IN in vitro. Autoradiography has revealed no significant change in 3′P (Fig. 3c) or STA (Fig. 3d) on varying the PSF protein concentration. The non-significant change in the activity of IN suggested the involvement of other cellular factor in the formation of stable synaptic complex once the IN binds to viral LTR ends.

### PSF negatively regulates the HIV-1 replication

**Fig. 4:**
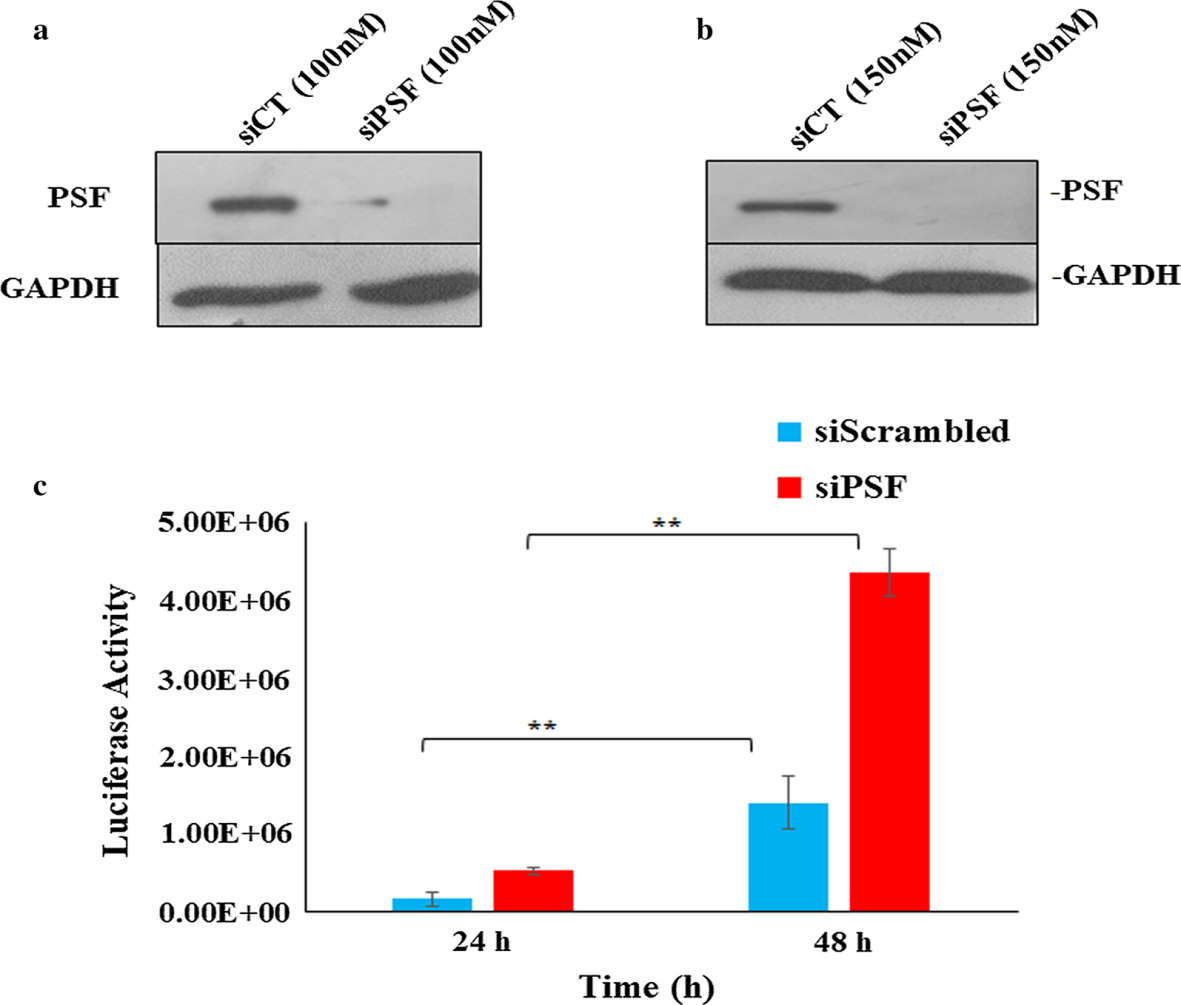
Impact of knockdown of PSF on HIV replication as measured by luciferase reporter gene assay. **a**, **b** are siRNA knockdown study at 100 and 150 nM respectively to achieve maximum knockdown. **c** Luciferase activity observed at 24 and 48 h at 1 MOI. TZM-bl cells were transfected with a pool of PSF siRNA (siPSF) at 150 nM and control siRNA (siScrambled) and viral replication was monitored. Data depicted here shows average values ± SD of 3 independent experiment (**p < 0.02). Knockdown was measured at every step

To inspect the role of PSF in HIV-1 replication, we performed the siRNA knockdown (KD) study. The cells were devoid of PSF (siPSF) using pool of siRNA and observe it’s susceptibility to viral infection using HIV-1 virus in TZM-bl cell line infected at 1 MOI. The siRNA was used at different concentration first to check the reduction in expression of PSF by western blot (Fig. 4a). At 100 nM we observed around 67–70% of PSF knockdown. To achieve maximum knockdown we increased the siRNA concentration to 150 nM and got more than 90% knockdown. Thus 150 nM was used in all studies of viral assay without any cell death. The unspecific siRNA (siScrambled or siCT) was used as a control. The viral replication was monitored at different time points by luciferase reporter gene assay. Viral assay demonstrated an increase in viral production or the cells were more susceptible to infection in PSF knockdown (siPSF) cells as compared to scrambled control cells at 1 MOI at both 24 and 48 h (Fig. 4c) (Paired t test yielded *p* value < 0.02). To exclude the off-target effect and the toxicity because of non-specificity, cell density was determined using cell counter after transient knockdown with siRNA at different time interval. PSF has been involved in DNA repair and the cells that are defective in DNA repair were proposed to undergo apoptosis as result of viral infection [30]. To examine the possibility of apoptosis, PSF knockdown and scrambled cell line was infected with HIV-1 virus and cell death was determined by trypan blue staining (Additional file 2: Fig. S2). Transduced and non-transduced cell line showed little or no effect in the apoptosis response till 48 h. Thus the difference in the viral response was not due to apoptosis.

The change in transduction efficiency in PSF knockdown cells instigated us to perform the viral infection in the PSF overexpressed cells by transfection with GFP-PSF plasmid. GFP only was used as a control. The plasmid transfection was visualized by Olympus fluorescence inverted microscope (Additional file 3: Fig. S3). The transfection efficiency and the cell viability was determined by fluorescence activated cell sorting (FACS) and MTT [31] assay (Additional file 4: Fig. S4 and Additional file 5: Fig. S5 respectively). The viral infection was reduced in PSF overexpressed cells as compared to its respective control (Additional file 6: Fig. S6) (Paired t test yielded *p* value at 0.1 and 0.5 MOI < 0.05) which is in accordance with our knockdown data but we also observed cell death after virus infection in both PSF overexpressed and it’s GFP only control cells in TZM-bl cell line. However FACS and MTT analysis has revealed no cell death after transfection with the GFP-PSF or GFP only plasmid.

To investigate it’s mechanism of action if the difference in viral infection or the enhanced susceptibility to infection was due to impairment of viral DNA integration or if the process is following some other pathway, we analysed the different HIV-1 DNA forms such as unintegrated DNA (2-LTR), late reverse transcription product (cDNA) and the integrated provirus at various time interval by quantitative real time PCR (qPCR) assay.

### Detection of reverse transcription products and integration events in PSF deficient cells

**Fig. 5:**
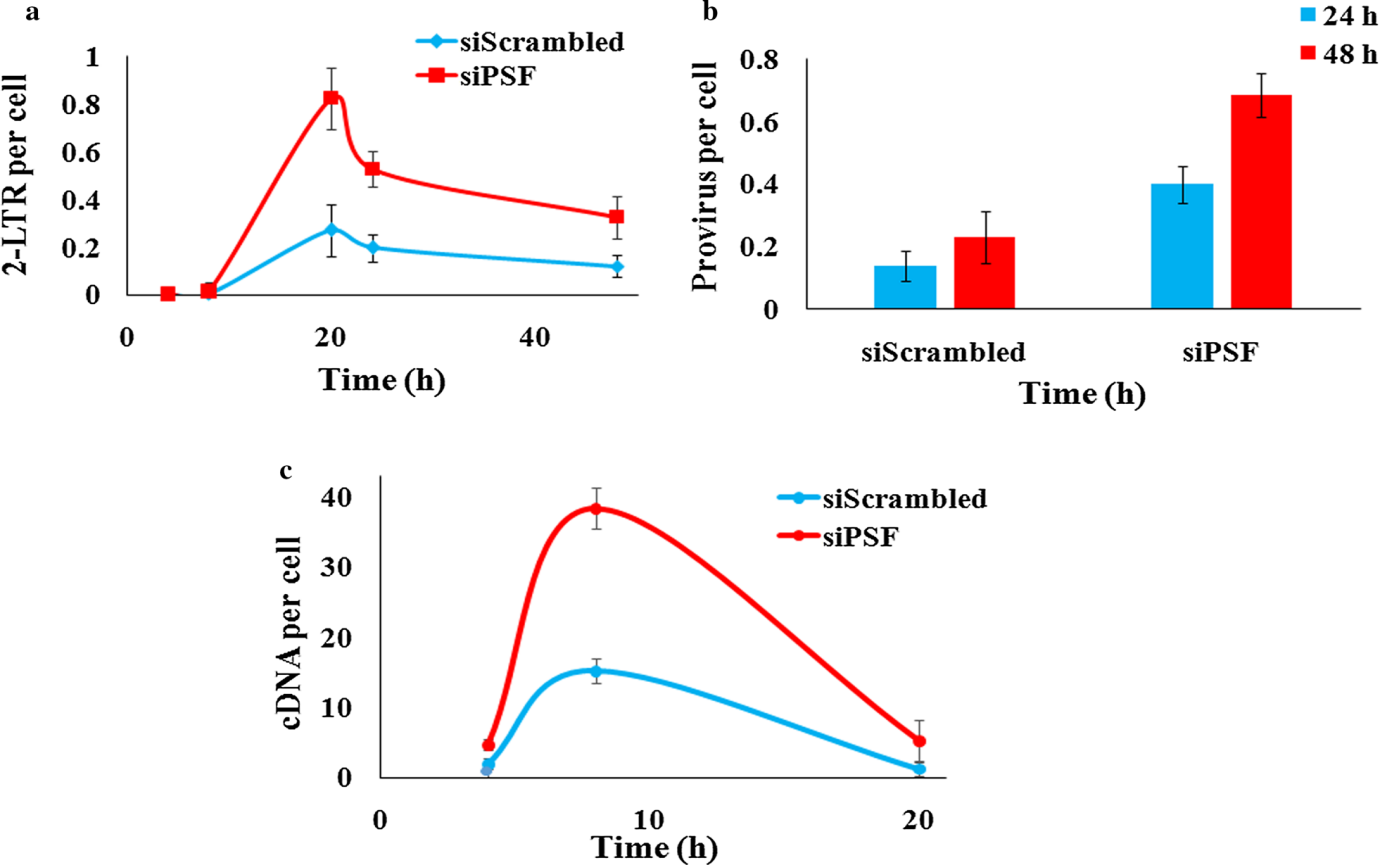
Quantitative PCR to analyse the impact of PSF knockdown on HIV-1 2-LTR, integrated provirus and late RT products. TZM-bl cell line was transduced with the pNL4-3 at 1 MOI. **a** 2-LTR detection at different time point in the PSF (siPSF) knockdown and control cells (siScrambled). Paired t test analysis revealed *p* values < 0.03. **b** Integrated provirus was detected by nested Alu PCR in PSF knockdown cells. Paired t test analysis revealed *p* values < 0.04. Error bar depicts SD between three independent experiment. **c** Viral cDNA was detected by qPCR at different time points. Error bar shows the SD between three independent experiment (*p < 0.05)

The production of different viral DNA forms was quantitated by qPCR. The specific primers and sybr green was used to detect 2-LTR, late RT products i.e. the full length cDNA molecules and the integrated provirus. The absolute quantification of formation of 2-LTR circle or the cDNA, was done by preparing the dilution of the respective plasmid to generate standard curve and then quantitating the unknown DNA sample. The PSF knockdown TZM-bl cells were infected with the virus at 1 MOI and the kinetics of different viral DNA forms was monitored. We observed that the 2-LTR circle DNA formation was twofold higher in PSF knockdown cells as compared to scrambled control cells. The 2-LTR DNA has reached to its peak level till 20–22 h and the difference was greatest at 20 h between knockdown (siPSF) and control cell (siScrambled) and then started declining (Fig. 5a) (paired t test *p* value < 0.03). We have also not observed any cell death till 48 h in the knockdown and control cells analyzed by trypan blue staining (Additional file 2: Fig. S2). The observed change in the 2-LTR formation provoked us to examine the viral cDNA level also. To evaluate the levels of viral cDNA, DNA was harvested at 2, 4, 8 and 20 h and virus late reverse transcription products were quantitated. The qPCR data revealed that quantity of cDNA was more in knockdown cells (siPSF) as compared to control cells (siScrambled) at 8 h and declines thereafter (Fig. 5c). In addition, to identify whether the increase in viral replication was due to modulation of stable integration events, the nested Alu PCR was done for detection of integrated provirus. The qPCR data revealed more integrated viral DNA in knockdown cells (siPSF) as compared to scrambled control cells (siScrambled) both at 24 and 48 h (t test *p* value < 0.04) (Fig. 5b). Thus the reduced PSF expression by siRNA knockdown modulates the cDNA metabolism or affects it’s stability and directly or indirectly also influenced the integration events. The quantitative PCR in PSF overexpressed cells detected decrease in 2-LTR, integration events and viral cDNA (Additional file 7: Fig. S7).

**Fig. 6:**
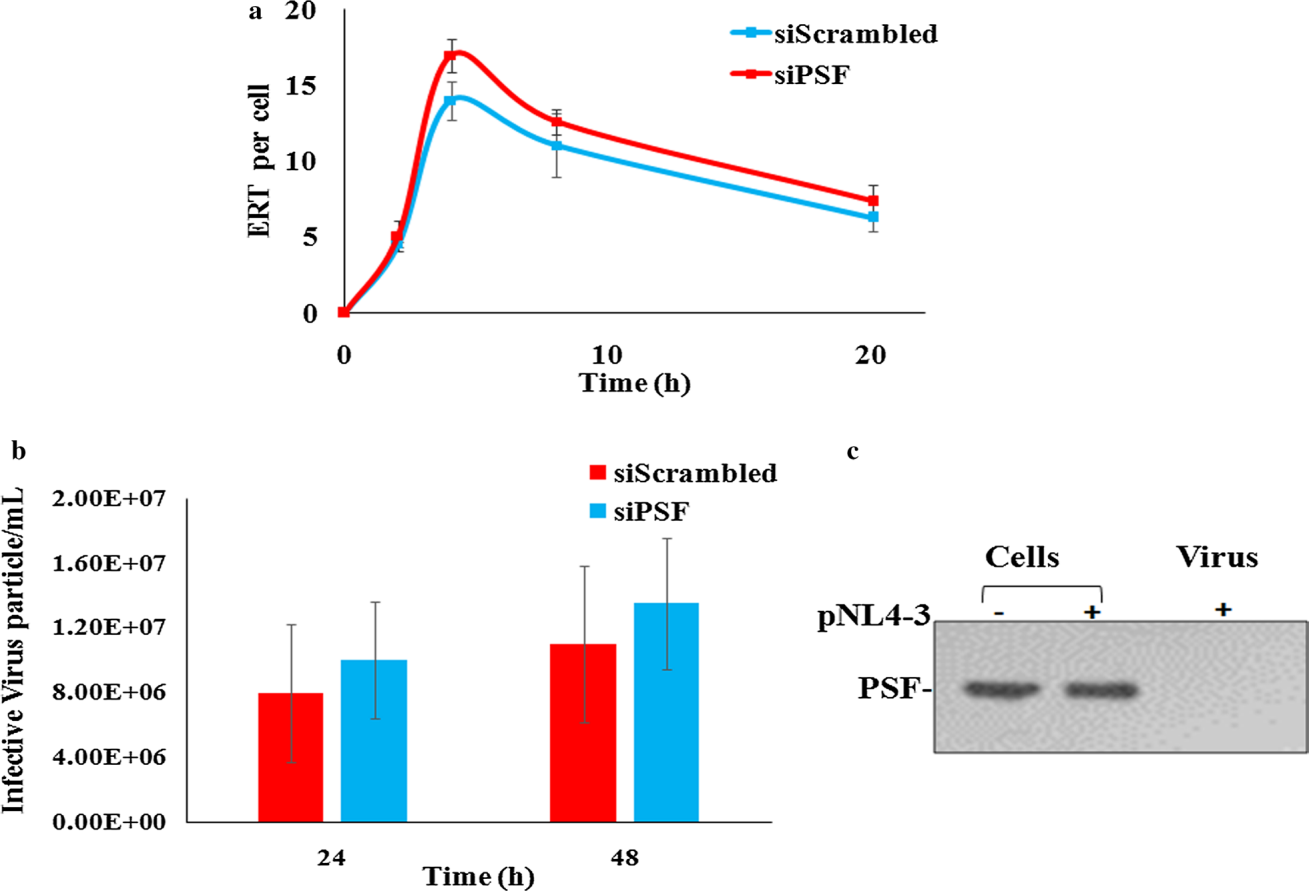
Evaluating the impact of PSF on different steps of virus life cycle. **a** Quantitative PCR to detect the formation of early RT product at different time point. Error bar depicts SD between three independent experiment (*p *> 0.05). **b** To examine the virus infectivity produced in PSF knockdown cells—Supernatant collected from PSF knockdown (siPSF) and scrambled (siScambled) cells were used to infect the TZM-bl cell line. The infective virus particle were calculated. No significant difference in virus infectivity was observed. Data depicted here shows average values ± SD of 3 independent experiment (*p *> 0.05). **c** Western blot to detect the PSF incorporation in the cells and virus of control cells by using 150 ng of protein and PSF was detected by anti-PSF antibody

The cellular proteins have already been implicated during reverse transcription. After the detection of modulated expression of late reverse transcript product, we suspected if PSF was acting as a cofactor during reverse transcription. To examine, if the difference in virus production in knockdown cells could be due to its role during the formation of reverse transcription products, we checked the level of early reverse transcript product. The TZM-bl cells were infected at 1 MOI and the formation of early reverse transcription products (ERT) by real time PCR was detected at different time points. The pNL4-3 plasmid was used to generate the standard curve. Here we did not observe significant difference in ERT products in PSF knockdown and control cells (t test *p* value > 0.05) (Fig. 6a). The result thus support that PSF is acting at cDNA level when complete viral DNA has been formed. The PSF and its domain interaction with viral cDNA is our ongoing studies to get the more clear view.

### PSF is not affecting the late steps of HIV-1 life cycle subsequent to integration

As PSF has been previously reported in viral gene regulation such as rev dependent export of viral RNA as well as interaction with INS containing mRNA and its inhibition, it led us to analyze the other viral replication stages. To check whether the PSF mediated negative regulation affect the infectivity of virus, we collected the supernatants from the virus infected PSF knockdown cells. The viral particles were counted by β-galactosidase assay and equal amount of viral particles from the PSF knockdown and control cells were used to infect TZM-bl cell line. The infected TZM-bl cells were grown till 48 h and then cells were treated with β galactosidase substrate to count the infective viral particles. No significant difference in the number of infected cells was detected between PSF expressing and PSF knockdown cells as quantified by β-galactosidase assay (Fig. 6b).

In addition to it, the proteins were also extracted from the same virus aliquot as well as from the cells producing normal amount of PSF to check the incorporation of PSF in the virus. We did not observe PSF incorporation in viral particles. Thus, it is unlikely to play any role during later stages of virus such as budding or maturation of virus particles and seems to act in the target cells only after virus entry (Fig. 6c).

### Plausible binding site elucidation of full length HIV-1 IN with PSF and viral DNA from molecular docking and MD simulations

The HIV-1 integrase has been proved to bind as a dimer with one DNA molecule. The negative charge on DNA interacts with several positive charges on integrase surface and thus providing the favorable binding pocket. The important amino acid residues of IN which were earlier reported [32] in binding to viral DNA are K156, K159, K160, K186, K188 of the CCD domain and S230, R231, W243, K244, R263, K264 of CTD domain. These binding helps in proper positioning of DNA during integration process. PSF belongs to Drosophila behavior/Human Splicing (DBHS) family that possess a very dissimilar RNA recognition motif (RRM) domain consisting of RRM1 from 299 to 369 amino acid residues, and RRM2 from 370 to 449 residues, NOPS domain (450–498 residues) and coiled coil domain (499–598 residues). The RRM is previously characterized nucleic acid binding domain. We have tried to understand the interaction of PSF with viral DNA and IN through computational approach.

**Fig. 7:**
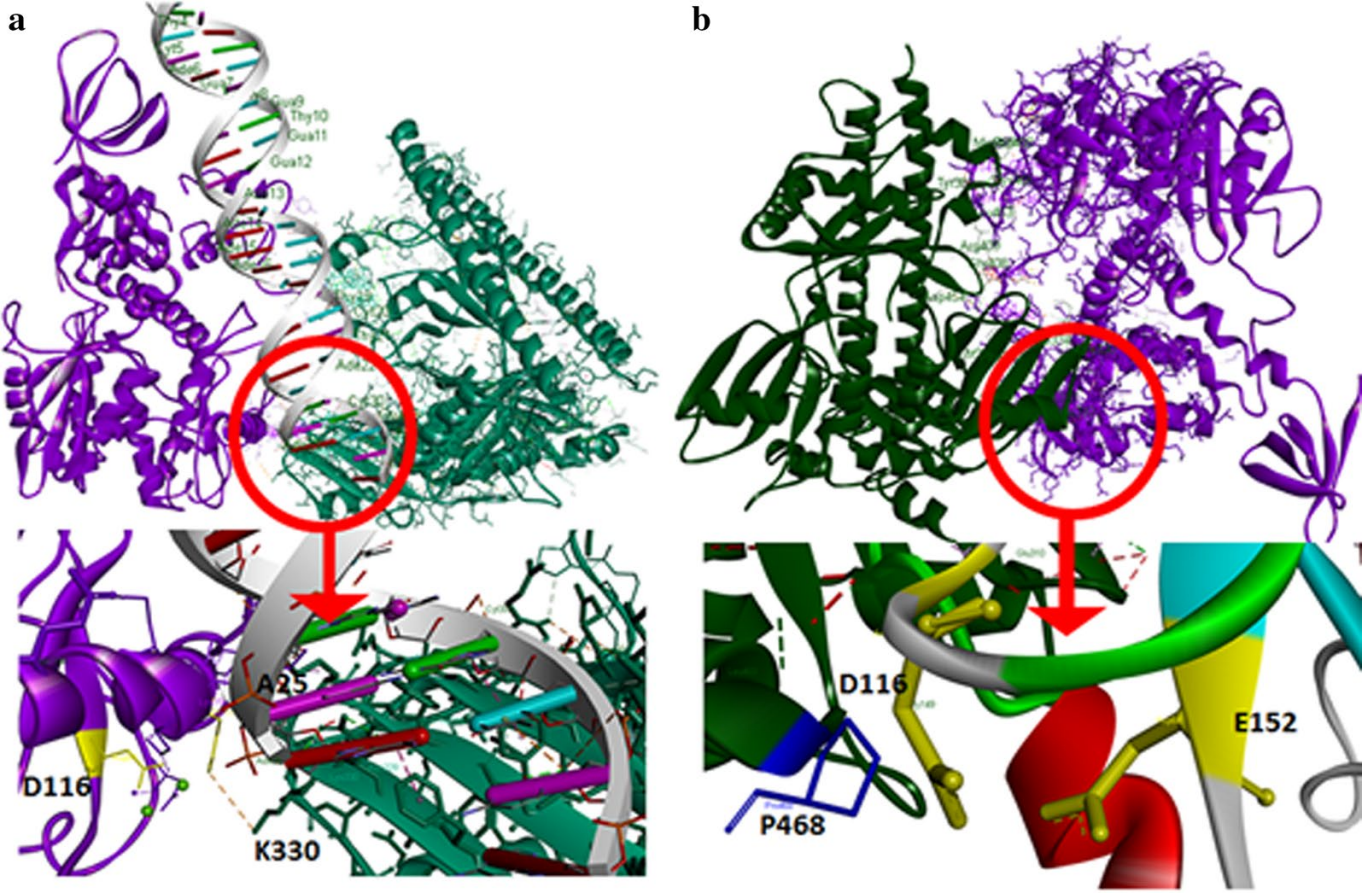
Docking structure of **a** full length HIV-1 Integrase (in green)-dsDNA (in stick) complex with PSF (in purple), with inset of DDE motif of CCD-domain, showing different residues of PSF and HIV-1 interacting with dsDNA, **b** full length HIV-1 Integrase (in green) with PSF (in purple), in inset, the DDE motif was found to be occupied with other residues, here D386 (in yellow stick) of PSF presents near D64 of HIV IN directly interacts with Mg^+2^ ions

In order to understand the mode of action by PSF, the molecular docking study of PSF with HIV-1 integrase and with HIV IN–DNA complex was performed. First, we have done molecular docking study of full length IN (containing 1–288 amino acid residues) and viral dsDNA (Additional file 8: Fig. S8). Our study has depicted conserved interactions of IN–DNA as reported earlier [31, 32]. The terminal site residue of viral DNA has been observed to be important during integration process by IN. It was found near DDE catalytic site triad (i.e. acidic triad D64, D116 and E152) of IN along with interaction of important residues like K156 and K159 via van der waal’s force to the terminal residues C30 and A25 of DNA respectively. Other important residues like K186, K188 and few residues of N-terminal domain have also shown interaction with the viral DNA. We performed the docking of binary complex of full length HIV-1 integrase-dsDNA with PSF that results in formation of ternary complex (Fig. 7a). The study provided strong evidence of the interaction of PSF with viral DNA also. K466 of PSF interacted with IN residues E146 by salt bridge interaction. Besides, E152 and W19 residue of IN interacts with P468 and K332 by charge interaction and van der waal’s interaction respectively. Along with the above, F300, K332, R360 and T368 of RRM1 domain of PSF was found to interact with phosphate backbone of DNA. The terminal adenine base (A25) of viral DNA was observed in interaction via it’s –NH with lone pair of oxygen of N329 of PSF forming a hydrogen bond (Table 3). While another residue F327 interacts with terminal adenine base A25 via π–π interaction. Residues belonging to RRM2 domain of PSF i.e. A370, R409, were observed to interact with T28 and C20 bases of viral DNA. D386 of PSF was found to interact with D64 of HIV-IN through Van der Waal’s interaction which hinders the possible DNA interactions (Fig. 7a). Besides that E152 was found to slightly displaced from Mg^+2^-ions, thus weakens its interaction and reduces the activity by IN catalytic domain and therefore supports our hypothesis. PSF residues 366–421 of RRM1 and RRM2 domain was observed near A26-C30 bases and residues 464–470 of NOPS domain was identified near C18-T19 bases.

**Table 3:** Interacting Residues of PSF with viral dsDNA and Integrase

PSF with binary complex of HIV-1 IN–viral dsDNA		PSF with HIV-1 IN	
AA* of PSF	DsDNA	AA* of PSF	AA* of HIV-1 integrase
K332	PO_4_-backbone	F300	M50
F300	PO_4_-backbone	E310	Y15
F327	A25	V326	H16
N329	A25	F327	H16
F334	C30	K330	N18
		D386	D64
		D454	H51
		K332	W19
R360	PO_4_-backbone	F334	A49
		T368	Q48
T368	PO_4_-backbone	Y381	I141
A370	T28	Y381	Q148
R409	C20	G408	V54
E465	T19	R409	V54
		D454	H51
		L463	Y143
		K466	E146
		N467	S147
		P468	E152
		R409	P145

*AA amino acid residue

After confirming the interaction of the ternary complex of PSF with integrase-DNA complex, another set of docking study was performed between PSF and full-length HIV integrase. The docked structure of PSF with IN (Fig. 7b) depicted few important interactions of Y143 and Q148 residues of IN with PSF. The Y143, Q148 has been reported in binding to viral LTR DNA. The hydroxyl group of Y143 of IN was observed to interact through hydrogen bonding with ‘NH_2_’ group of L463 of PSF. The ‘–NH’ of Q148 of IN formed hydrogen bond with ‘OH’ group of Y381 of PSF. The flexible loop region [33] residues (140–149) of IN, P145, E146 and S147 interact with R409, K466, N467 of PSF respectively via van der waal’s force (Table 3). The other residues of IN, A49, V54 and I141 which is already known to interacts with viral DNA [34] were also observed to interact with residues of RRM1 and RRM2 domain of PSF. The residues 450–460 of monomer A subunit of PSF was found near IN region 150–160 of CCD domain while the region 300–334 of monomer subunit B of PSF was found near 15–50 region of N-terminal domain of IN.

After triplex conformer elucidation from molecular docking, a reasonable 100 ns MD simulation was performed for better structural conformational study to confirm the mode of PSF binding to IN–viral DNA complex. A considerable amount of MD produced better structural information with binding energy calculation in between of HIV IN, PSF and dsDNA. From MD trajectory analysis, we found HIV IN and PSF bound each other very firmly as compared to the dsDNA. PSF was found to interact with the invariant bases of viral DNA and with IN residues that plays significant role in integration (see Additional file 9: MD Simulation movie clip). The lower binding free energy, − 29 kcal mol^−1^ was found with dsDNA to HIV IN–PSF complex (Table 4), whereas a higher − 41.01 kcal mol^−1^ was found in between HIV IN and PSF. The number of H-bonds found to be remain constant during 100 ns time span also revealed the efficient binding of PSF with HIV IN (Additional file 10: Fig. S9A). All atoms-RMSD curve of dsDNA depicted a bit fluctuation after 50 ns (near 50–70 ns) and the curve is showing a trend to increase its fluctuation till 100 ns of dsDNA complex in ternary complex of HIV IN–PSF–dsDNA (Additional file 10: Fig. S9B). The RMSD is showing that our MD was successfully converged with a RMSD of 0.08–0.1 Å, even though a huge number of amino acid residues being present (a total of 1042 residues). These regions and neighboring dsDNA exhibit high flexibility, possibly explaining their less association in ternary complex. The binding pockets which found after HIV IN–dsDNA docking was tends to got occupied by PSF mostly, replacing backbone interactions of dsDNA. Herein, we have reported first time MD simulation of the ternary complex of HIV IN–dsDNA–PSF and found a supportive mechanistic approach which can help to study in detail further.

**Table 4:** Binding energy calculation from MD trajectory of HIV IN–dsDNA–PSF ternary complex

Energy	Between HIV IN and PSF	Between HIV IN–PSF complex with dsDNA	Between HIV IN–dsDNA complex and PSF
VDWAALS	− 104.58 ± 05.42	− 124.96 ± 7.34	− 200.52 ± 07.42
EEL	− 382.26 ± 28.83	− 2437.34 ± 128.29	− 98.61 ± 59.68
ΔG (gas)	− 444.72 ± 25.98	− 2562.31 ± 125.90	− 299.14 ± 59.26
ΔG (Total)	− 41.01 ± 04.53	− 29.60 ± 09.13	− 34.60 ± 13.65

All energies are represented in kcal mol^−1^

## Discussion

In this study our goal was to identify the new cellular interacting protein of HIV-1 IN, to understand the mechanism of it’s action in HIV-1 replication and subsequent development of new therapeutic approaches. Multiple studies have shown the interaction of PSF in viral replication. We investigated the cellular multifunctional protein PSF interaction with HIV-1 IN and its effect on HIV-1 replication. Through pull down, co-IP and purified protein interaction assay we demonstrated the direct physical interaction between IN and PSF. The colocalization study further confirm the interaction between the two proteins. Though, the p54nrb/PSF has already been reported to downregulates the viral mRNA mediated via INS, the findings observed in our study correlated to PSF mediated downregulation of HIV-1 integration invoked us for an alternative hypothesis in order to understand the phenomena. The knockdown study of PSF has identified it to negatively regulates HIV-1 replication in mammalian cell line. The 2-LTR DNA circle analysis which requires NHEJ repair shows that it’s formation can be affected by PSF expression. Moreover, quantitative PCR assay detected higher level of late reverse transcript or viral cDNA and viral integration events in PSF knockdown cells in early hours. The changes at cDNA level suspected the involvement of PSF at reverse transcription process. To clarify it further we analysed early reverse transcription product by qPCR also but we did not observe significant changes in control and knockdown cells. PSF are known to bind to both DNA and RNAs. Here the significant difference observed in late reverse transcription products rather than early reverse transcript suggested that PSF has greater role to play at the late phase of reverse transcription when complete viral DNA has been formed rather than to the viral RNA. Thus it is possible that PSF is contributing during late phase of reverse transcription by DNA binding or DNA–protein binding mechanism thus supports our hypothesis of binding of PSF to IN and viral-dsDNA complex. Our in vitro integrase activity assay in the presence of PSF did not shows any change in the activity. This could be due to post translational modification occurring in PSF inside the cell during the viral infection or PSF must be acting collaboratly along with some other cellular cofactors and affecting viral replication.

PSF has been demonstrated by various studies to impact not only RNA biogenesis but also plays role in several DNA mediated process. It has direct role in DNA damage response, particularly in recognition and repair of DNA double strand break (DSB). PSF binds to DSB through part of its RGG box encompassing proline rich domain. The same domain is also involved in interaction of PSF with RAD51 which plays important role in homologous recombination (HR) [26]. RAD51 protein binds to single or double stranded DNA and plays important role in HR and is already reported to interact with HIV-1 IN and inhibits the integration by remodeling the IN–DNA complex and thus dissociation of retroviral enzyme from substrate [35].

We have used the computational approach to elucidate the binding site determination affecting HIV-1 replication by PSF. The study of ternary complex of dsDNA-IN–PSF suggested the interaction of PSF with the viral DNA ends as well as with the important residues of IN which significantly interacts with viral DNA and required for proper positioning of viral DNA and its integration. PSF RRM1, RRM2 and few residues of NOPS domain was found to interacts with the flexible loop region residues 140–149 of IN protein. The residues spanning the flexible loop region of IN is present adjacent to the catalytic triad residue DDE of IN which is crucial in catalysis process and helps in proper positioning of viral DNA. The computational study identified the interaction of ends of viral DNA A25 with PSF region which is required in catalysis (Table 3). The extensive contact of PSF with the invariant conserved sites of viral DNA ends suggests it’s preference for binding and inhibition of viral IN inbound form with DNA.

## Conclusion

PSF plays multifaceted role inside the cell. Our study has provoked us to propose an alternative hypothesis that the negative impact of PSF on viral replication was due to binding of PSF protein to integrase-HIV-1 cDNA complex and ultimately destabilizing the complex which leads to decline in the number of integration events. PSF was not found to influence the late steps of viral replication as well as during the formation of early reverse transcript product. Here the downregulation was observed which can be correlated to the decrease in the number of stable integration as we did not observe cell death after PSF knockdown or after HIV-1 infection. Although we cannot rule out the possibility that PSF may be preventing the interaction of other proteins which are involved in binding with integrase or cDNA or the association of other retroviral proteins binding to cDNA thus preventing the overall HIV-1 integration.

Many mechanism of action is still not clear but the outcome from the data suggested the involvement of PSF in virus life cycle. The mechanistic approach involved to study the downregulation of HIV-1 replication illustrated in our study and the newer assays to detect domain wise interaction of PSF with HIV-1 IN is under active study. This could lead to the improvement of such therapeutic strategy further.

## Methods

### Materials procured

All materials were purchased from sigma if not indicated otherwise. Antibody against IN was purchased from Santa cruz, sc-69721, anti-PSF (P2860) and anti-His (SAB1305538-40TST) antibody purchased from Sigma, horseradish peroxidase (HRP) conjugated goat anti-mouse secondary antibody was purchased from Santa cruz-sc2005.

### Protein binding assay

The His-IN protein was purified by Ni–NTA affinity chromatography. The His-IN plasmid was transformed in *E. Coli* BL21. It is then grown in 1 L of Luria Broth medium in presence of 100 µg/mL of ampicillin till absorbance reaches to 0.6 at 600 nm wavelength. Protein was induced with IPTG at a final concentration of 1 mM. Cells were harvested after 4 h, resuspended and lysed in buffer A containing 20 mM HEPES, pH 7.4, 1 M NaCl, 5 mM imidazole, non-ionic detergent Chaps. After 30 min incubation on ice, cells were sonicated, treated with DNase, centrifuged at 10,000×*g* for 30 min. The supernatant was applied to the charged Ni–NTA Sepharose affinity column (Qiagen) and washed with buffers containing increasing concentration of imidazoles, from 20 to 60 mM and the final elution with 300 mM imidazole. The IN protein fractions were then pooled and purified through G25 Sephadex column chromatography with buffer containing 20 mM HEPES, 0.5 M NaCl, 1 mM DTT and 10% glycerol. It is then stored at − 80 °C.

His-IN was diluted to a final concentration of 500 ng in the binding buffer 20 mM Tris–Cl (pH 7.4), 0.1 M NaCl, 5 mM MgCl_2_, 0.1% NP-40, 200 mM phenyl methyl sulphonyl fluoride (PMSF), 20% glycerol (pH 7.4). The protein was centrifuged at 16,000*g* for 15 min and transferred to a fresh tube. The resin or slurry 50 µL was added to the His-IN after washing the resin with binding buffer. 1 µg of HeLa cell lysate protein was collected in another tube in a binding buffer as described earlier for 4 h at 4 °C. Both the proteins were incubated with micrococcal nuclease enzyme (MNase) (NEB) for 10 min at 30 °C to eliminate the contamination with nucleic acid as described previously [36]. Both the proteins containing beads were mixed and incubated for 3 h at 4 °C on a rotatory shaker. The mixture were centrifuged at 800 g for 2 min, supernatant was removed. The beads were washed with 500 µL of binding buffer. The protein sample was then eluted in 1x SDS sample by heating at 95 °C for 2 min and analysed on 10% SDS–Polyacrylamide gel electrophoresis (PAGE) gel by staining with coomassie R250 staining solution. The interacted protein bands were cut from the gel, and collected in a tube. The band were destained in an ammonium bicarbonate solution (200 mM), 50% acetonitrile. It is air dried and incubated with 8 µL of trypsin gold (Promega) solution (16 ng) in 50 mM ammonium bicarbonate for 30 min on ice. It is then incubated overnight at 37 °C and the supernatants were collected. The gel pieces were extracted again with 60% acetonitrile and 0.1% formic acid. The extracted protein and supernatant were pooled. Speedvac concentrator were used to lyophilize the protein. The peptides were then dissolved in 0.1% formic acid and injected in nanoflow high performance liquid chromatography coupled with a Q-Tof mass spectrometer and equipped with an electrospray ionization source (LC/MS/MS). The data and spectra (Additional file 11: Fig. S10–S14 and Fig. 1b) were analysed in a Proteinpilot software, SCIEX.

### Cell lines and plasmids

Genetically engineered TZM-bl cells that express galactosidase and Luciferase under the influence of HIV-1 LTR were procured from NIH, USA under the AIDS Research Reference Reagent Program (ARRRP) [37] HeLa, HEK 293T and TZM-bl cell line were cultured in Dulbecco’s modified eagle’s medium (DMEM) with 10% fetal bovine serum and 1% antibiotic antimycotic solution (Invitrogen) containing penicillin, streptomycin, and amphotericin in 5% humidified CO_2_ atmosphere at 37 °C. GFP-PSF plasmid (eGFP-C1 vector) was a kind gift from Barbara K. Felber, National Cancer Institute Frederick, Alessandro Marcello, International Centre for Genetic Engineering and Biotechnology (ICGEB), Trieste, Italy and mRFP-IN plasmid was a gift from Jan De Rijck, Department of Pharmaceutical and Pharmacological Sciences, KU Leuven, Belgium. The bacterially expressed plasmid of IN, pINSD.His.sol was a kind gift from Dr. Raymond Heuwer, South Africa. The standard control for 2-LTR was a kind gift from Dr. Kristine Yoder, Ohio state University, ACH-2 cell line as a standard plasmid for integrated provirus detection was obtained from NIH, USA under the ARRRP [38]. (For viral late reverse transcripts (LRT) product or cDNA, gene was cloned in pGEM-T vector. The primers for LRT was used as described by Yoder et al. [39] Fp 5′GCTTGCCTGCAGTGCTCAAA3′, Rp 5′TGCCGTGAGCTCTTCAGCAA-3′. The gene was amplified using pNL4-3 as template. The amplified product and vector was digested at Pst1 and Sac1 (HF) site and ligated by T4 DNA ligase. The plasmid was confirmed through sequencing.

### Co-immunoprecipitation (co-IP) and western blot

The HeLa cells were seeded overnight before transfection with mRFP tagged IN. The cells were harvested after 24 h and lysis was done in 0.25% Nonidet (P-40) buffer. The supernatant (500 µg/mL) were collected after centrifugation at 12,000*g* for 30 min at 4 °C. It is then treated with MNase enzyme. The protein quality was analysed on SDS PAGE gel. The nuclear protein were then incubated with anti-IN antibody bound protein A/G agarose beads for 4 h at 4 °C with gentle rocking. The complex was eluted and subjected to SDS–PAGE gel electrophoresis as described earlier [40]. The proteins were then transferred to nitrocellulose membrane (MDI), treated with anti IN and anti-PSF antibody. HRP conjugate anti-mouse antibody was used as secondary antibody. The visualization of protein was done by ECL substrate kit (Thermofischer).

### Purified protein–protein interaction

In vitro direct physical interaction between the two proteins was identified by using purified proteins. To confirm the interaction, both his tagged proteins were purified by Ni^+2^–NTA affinity chromatography after DNAse treatment. His tag of IN was completely removed using 100 µL of thrombin-agarose resin (thrombin clean cleave kit, Sigma) following the manufacturer’s recommendation for 12 h at 30 °C. The 20 µL aliquot was taken at 2, 4, 8 and 12 h to check the His cleavage. The removal of Histidine tag at 12 h was confirmed by western blot using anti-His and anti-IN antibody (Additional file 1: Fig S1). The IN protein was then dialyzed in 20 mM Tris–Cl, 200 mM NaCl. For the interaction study, 500 ng of His tagged PSF was immobilized on Ni^+2^–NTA agarose beads followed by incubation with 500 ng of IN for 4 h. The complexes recovered through the bead was resolved by SDS PAGE and analyzed by western blot.

### Cell transfection and study by fluorescence microscopy

HeLa and HEK 293T cell line were seeded on a cover slip in six well plate and transfected with GFP-PSF plasmid and monomeric Red Fluorescent protein tagged IN (mRFP-IN) in 1:1 ratio (800 ng) using lipofectamine 2000 (Invitrogen) in a serum free antibiotic free medium. After 24 h, cells were washed with phosphate buffer saline (PBS) and fixed with 4% paraformaldehyde. It is quenched with 0.1 M glycine and permeabilised with 0.1% between 20. After washing with 3X PBS, the nuclear DNA was stained with DAPI (50 ng/mL) diluted in PBS for 5 min. The cover slip were mounted in a mounting medium and then cells were observed under Olympus fluorescence inverted microscope (TH4-200, Tokyo, Japan). GFP was observed in a FITC filter and mRFP under TRITC filter.

The overexpression of PSF was done with GFP-PSF plasmid in TZM-bl cell line following the above protocol. Transfection efficiency was calculated in TZM-bl cells after transfection with 800 ng of GFP and GFP-PSF plasmid per well in a six well plate using lipofectamine 2000. It was harvested after 24 h washed with PBS and efficiency was calculated by Fluorescence activated cell sorting (FACS, BD LSR Fortesa), using FITC filter (Additional file 4: Fig. S4).

### Cell viability assay

The cell survival was determined by 3-(4,5 Dimethylthiazol-2-yl)-2,5 diphenyltetrazolium bromide) (MTT) reagent—Ten thousand TZM-bl cell line was seeded in 96 well plate, and transfected with GFP-PSF and GFP plasmid at concentration, 20 ng, 50 ng, 100 ng, 150 ng using lipofectamine 2000 in a serum free antibiotic free media. After 24, 48 and 72 h, MTT reagent 20 µL per well (5 mg/mL) were added. The insoluble formazan crystal were dissolved in DMSO. Percentage of cell survival was determined after reading absorbance at 570 nm wavelength (630 nM reference wavelength) (Additional file 5: Fig S5).

### Cloning of PSF in bacterial expression vector

The cloning of PSF in bacterial expression vector pPROEX-HTC was done using GFP-PSF as a template. The primers used for cloning was FP 5′TCA AGC TTC GAG CTC TGC AGC TTG ACC AC 3′, RP 5′CAA ACT GGA ATG AAA GCC TAG GTA CCA CAT CTA AAAT 3′. A 20 µL PCR reaction consists of 1× GC buffer, 2 mM dNTPs, 10 ng template, 0.2 µL pfu polymerase. The PCR conditions were 95 °C for 5 min, annealing at 60 °C for 1 min, extension at 72 °C for 2 min for 30 cycles. The amplified PCR product were purified by PCR/gel purification kit (Qiagen). The vector 5 µg pPROEX-HTC and the PCR product were digested with 5 µL of each Kpn1 and Sac1 high fidelity restriction enzyme in 1× cut smart buffer for 4 h. The digested band was run on 1% agarose gel electrophoresis. The plasmid band was cut and purified by gel extraction kit (Qiagen). The digested band was ligated with ligase enzyme (1 µL).

### Protein induction and purification

The wild type protein is purified from Ni to NTA Sepharose column chromatography. The plasmid pPROEX-HTC-PSF containing polyhistidine tag at amino terminal end of PSF gene is transformed in *E. coli* BL21 [41] and grown in 1 L of Luria Broth medium in presence of 100 µg/mL of ampicillin till OD reaches to 0.6–0.8 at 600 nm as described earlier. Protein was induced with isopropyl-1-thio-β-d-galactopyranoside (IPTG) at a final concentration of 1 mM. After induction cells were grown at 20 °C for 8 h. Cells were harvested after 8 h, frozen in liquid Nitrogen and stored in − 80 °C. The cells were resuspended and lysed in buffer A containing 20 mM HEPES, pH 7.4, 1 M NaCl, 5 mM imidazole, non-ionic detergent chaps. After 30 min incubation on ice, cells were sonicated and then centrifuged at 10,000×*g* for 30 min. The pellet were again dissolved in a urea buffer containing 8 M urea. It is again sonicated, centrifuged and the supernatant was applied to the charged Ni–NTA Sepharose affinity column (Qiagen). The column was then washed with buffers containing increasing concentration of imidazoles, from 20 to 60 mM and the final elution of protein with 300 mM imidazole in 1.5 mL tubes. The PSF protein fractions were pooled and dialysed sequentially using decreasing concentration of urea. It was stored at − 80 °C. The wild type integrase protein is purified from Ni–NTA Sepharose column chromatography. The His-IN clone containing polyhistidine tag at amino terminal end and F185 K, C280S substitution is transformed in *E*. *coli* BL21 [42–44] and purified as discussed above.

### In vitro activity assay

The 3′P assay was performed with 21 mer oligos 5′ATGTGGAAAATCTCTAGCAGT 3′, 5′ACTGCT AGAGATTTTCCACAT 3′. For STA 19 mer oligos used were: 5′ATGTGGAAAATCTCTA GCA 3′, 5′ACTGCTAGAGATTTTCCACAT3′ Target DNA, Oligos were: 5′TCGAGAAAAAAAA AACTTAAGCCCCCCCCCCC 3′, 5′TCGAGGGGGGGGGGGC TTAAGTTTTTTTTTTC 3′. One of the 3′P and STA oligos were first labelled at 5′end with [γ-^32^P] ATP with the help of polynucleotide kinase. The second unlabeled strand is then annealed to it. The unlabelled DNA were removed by column chromatography purification (Qiagen purification kit). Activity assay were performed as described earlier [45–48]. The 20 µL reaction mixture contains of 250 nM IN protein, 20 mM HEPES, 5 mM MgCl_2_, 100 mM NaCl, 5 mM DTT, and 0.1–1 µM of PSF protein. The reaction was incubated on ice and then at 37 °C for half an hour for 3′end processing activity. For strand transfer reaction after addition of labelled donar DNA, target DNA is added and incubated for 1 h at 37 °C. The reaction mixture was then loaded on 15% PAGE and electrophoresed in tris borate buffer, pH 8. The gels were visualized by phosphorimager.

### Transient knockdown of PSF

The pool of siRNA (Sigma) was used to knockdown human PSF [23]. The sense primer for siRNA against PSF (siPSF) were 5′ GAAGAAGCCUUUAGCCAAU 3′, 5′ GCAAAGGAUUCGGAUUUA 3′, 5′ GAACAAAUGAGGCGCCAAA 3′, 5′ GGGAAAGAACAUGCGAAU 3′. In parallel, siRNA (sigma) against unspecific gene were used as negative control called as scrambled siRNA (siScrambled or siCT). 4 × 10^5^ HEK 293T or TZM-bl cell line were seeded in six well plate and transfected with siRNA at 100 nMand 150 nM using lipofectamine 2000 (Invitrogen) without causing any change in cytotoxicity. After 48 h, cells were lysed and knockdown was detected by western blot. To exclude the off target effect, cell viability was determined after transient knockdown with siRNA. The cells were seeded 12–16 h prior to transfection with siRNA. After 24, 48 and 72 h, the knockdown cells were trypsinized and cell density was determined by haemocytometer.

### Virus production and transduction in knockdown cells

HEK 293T cell line were seeded at 4 × 10^5^ cells in a six well plate. After 12–16 h, cells were transfected with 3 µg of pNL4-3 plasmid using calcium phosphate. After 3 days, supernatant were collected, filtered through 0.45 micron filter, aliquoted and stored at − 80 °C. 50% tissue culture infectious dose known as TCID_50_ was determined as follows (software used-Luc software by Duke University). Briefly several fold dilution of one of the stored virus aliquot were prepared in 96 well plate. Ten thousand TZM-bl cells in DMEM media containing 80 µg/mL DEAE-dextran were added in each well and incubated at 37 °C in CO_2_ incubator. After 48 h detection was done by britelite plus Reporter Gene assay (PerkinElmer) System in a luminometer. For the viral infection PSF knockdown and siRNA scrambled cells were infected with virus at 1 multiplicity of infection (MOI) in TZM-bl cell in a DMEM media containing 80 µg/mL in a 24 well plate. After 2 h, media was replaced with fresh DMEM medium. The infected cells were detected for viral expression at 24 and 48 h [49–52] by microplate reader (Perkin Elmer Victor 3). The overexpression of PSF for viral infection study was done by GFP-PSF plasmid. GFP only (pEGFP-C1 vector) was used as a control. The infection of virus is done as above (Additional file 5: Fig S5).

### Quantitative real time PCR assay

The knockdown cells were infected with 1 MOI of virus and harvested at different time point (2, 4, 8, 20, 24, 48 h). The DNA was extracted using DNase blood and tissue extraction kit (Qiagen) and used for quantitative PCR analysis [53, 54]. The different viral DNA forms i.e. early reverse transcript, 2-LTR and integrated viral product was quantitated using sybr green. The primers used were—for 2-LTR-Fp-5′ GTGCCCGTCTGTTGTGTGACT-3′, Rp-5′ CTTGTCTTCTTTGGGAGAGAATTAGC-3′, for late RT, Fp-5′-TGT GTGCCCGTCTGTTGTGT-3′, Rp-5′-GAGTCCTGCGTCGAGAGAGC-3′. Integrated product was detected by nested Alu PCR. For 1st round-Fp-5′ GCCTCCCAAAGTGCTGGGATTACAG 3′, Rp-5′ GCTCTCGCACCCATCTCTCTCC 3′. For 2nd round Fp-5′ AGCTTGCCTTGAGTGCTTCAA 3′, Rp-5′-TGACTAAAAGGGTCTGAGGGATCT 3′. The DNA for qPCR was used at 150 ng for detection of 2-LTR and late RT product. Detection of provirus was done as described by Butler et al. [55], Yun et al. [56] 2008. 1st round was detected using 250 ng of DNA and 2 µL of first round was used in 2nd round real time assay [57–60]. The viral DNA was also detected in the PSF overexpressed TZM-bl cells following the similar methodology used in knockdown experiments. The primers used for early RT product detection were, Fp-5′TAACTAGGGAACCCACTG 3′, Rp-5′ GTCTGAGGGATCTCTAGTTAC 3′. DNA was harvested at 2, 4, 8, 20 h time point and 100 ng was used for ERT product quantitation.

### Infectivity assay of viral particles after PSF knockdown

Virus was produced in HEK 293T cell line after transfection of pNL4-3 plasmid by calcium phosphate method. For determining viral particle per ml β-gal assay was performed on TZM-bl cells. Ten thousand TZM-bl cells in DMEM media were added in each well and incubated at 37 °C in CO_2_ incubator. After 16 h cells were infected with stored supernatant of pNL4-3 virus with different dilutions for 4 h. After infection cells were washed with serum free media to remove unbound virus and fresh media was added for 36 h. After 36 h cells were fixed by 0.05% gluteraldehyde and washed subsequently with PBS. For performing β galactosidase (β-gal) assay on TZM-bl cells freshly made β-gal substrate solution was added on the cells after fixation and kept for 2–24 h. After incubation blue cells were counted and viral particle per ml was calculated as follows: (Number of blue cells * dilution factor * plate factor * 1000)/volume of supernatant added. The virus infectivity assay was done in TZM-bl cell line. The supernatant was collected from the virus infected PSF knockdown and control cells at different time points. 50X10^4^ TZM-bl cells were seeded in 24 well plate. Next day, it was infected with the equal number of virus particles calculated by β-gal assay. After 36 h cells were fixed by 0.05% gluteraldehyde and washed subsequently with PBS. For performing β-gal assay on TZM-bl cells, freshly made β-gal substrate solution was added on the cells after fixation and kept for 2–24 h.

### Statistical analysis

The data for luciferase assay and qPCR were analysed using paired t test that yielded two tail *P* value (GraphPad PRISM 7).

## Computational study

The fast Fourier transform (FFT) based rigid-body protein–protein docking algorithm, ZDOCK [61] was used to perform unbound docking. We downloaded the docking predictions from the ZDOCK website, which were obtained using ZDOCK 3.02 (http://zlab.bu.edu/zdock). The desired PDBs 1WKN [62] and 4WII [63] were obtained from the Protein Data Bank. The full length (1–288 amino acids) structure of HIV-1 Integrase was based on the published MD-solved structure 1WKN with 27 mer dsDNA (U5). Two different docking were performed; (1) docked dsDNA-HIV-1 Integrase complex with PSF, (2) free HIV-1 Integrase with PSF. During the docking procedure one protein is kept fixed, while the other was moved around the fixed one. A grid-based representations was generated from the full atom chains of receptor and ligand and after each ligand rotation the grids can be fast convoluted via FFT. The ZDOCK Server allows users to generate sets of predictions in PDB format from job output files. The top 10 models that are available as user download, sets of predicted complexes were generated using an executable file that is included with the download of the appropriate program; Discovery Studio [64] was used here to visualize and analyze the structure. All the docking study was validated with other available server as online docking softwares i.e. PatchDock as well as Swiss Dock. The DNA sequence used for docking 5′-TAGTCAGTGTGGAAAATCTCTAGCA-3′. GT base removed from the 3′end.

We used Amber 16 [65] for all MD simulations and free energy calculations. HF/6–31 G* level of theory was taken for ab initio calculation from Gaussian 03 and calculation of partial charges were done by the restraint electrostatic potential method. The whole system was neutralized with Na^+^ ions and taken into a rectangular box of TIP3P water extending over 10.0 Å from the quadruplex exterior. Ptraj module of Amber14 was used for post MD analysis [66]. VMD 1.6.7 was used to perform analysis of trajectories, [67] whereas trajectory visualization was done using Chimera-1.5 [68] graphical programs. The analysis of free energy from the production trajectories using the single trajectory MM-PBSA approach was used [69, 70]. The free energy difference of binding was measured with the following equation.

$$\Delta G_{Free} = \Delta G_{electrostatic} + \Delta G_{vdW} + \Delta G_{non - polar} + \Delta G_{polar} - T\Delta S$$

where *ΔG* and *ΔH* are the binding free energy and enthalpy change at temperature *T* respectively.

## Additional files

**Additional file 1: Figure S1.** Histidine tag cleavage of IN using Thrombin.

**Additional file 2: Figure S2.** Measurement of cell viability percent in PSF knockdown and scrambled control cells by trypan blue exclusion dye.

**Additional file 3: Figure S3.** Transfection of GFP-PSF and GFP plasmid was performed in HEK 293T and TZM-bl cell line using lipofectamine 2000 and observed under fluorescence microscope (Olympus).

**Additional file 4: Figure S4.** The transfection efficiency was calculated after transfection with the 1 µg plasmid and percentage transfection was calculated by Fluorescence activated cell sorting (FACS). [A] Untransfected cells were shown as P3. [B] Transfection with GFP only with P4 region depicting the transfected cells. [C] Transfection with GFP-PSF with P4 showing the transfected cells.

**Additional file 5: Figure S5.** Cell viability was determined by 3-(4, 5Dimethylthiazol-2-yl)-2, 5 diphenyltetrazolium bromide) (MTT) reagent. TZM-bl cells were transfected with respective plasmid at concentration from 20 ng to 150 ng per well in triplicates and the percent viability or survival was determined by MTT after 24, 48 and 72 h.

**Additional file 6: Figure S6.** Analysis of overexpression of PSF on HIV replication as measured by luciferase reporter gene assay. [A] & [B] are the luciferase activity at 24 and 48 h at 0.1 and 0.5 MOI. TZM-bl cells were transfected with GFP-PSF plasmid. GFP with the same backbone was used as a control and viral replication was monitored. Data depicted here shows average values ± SD of 3 independent experiment. (*p < 0.05).

**Additional file 7: Figure S7.** Quantitative PCR of HIV-1 2-LTR, integrated provirus and cDNA per cell—The TZM-bl cell line was overexpressed with the GFP-PSF plasmid and GFP was used as a control plasmid. Cells were transduced with the pNL4-3 virus at 0.5 MOI and DNA was detected by qPCR at different time points. [A] 2-LTR detection at different time point after overexpression of cell, infecting with pNL4-3 virus and harvesting DNA for analysis. [B] Integrated provirus was detected by Alu nested PCR at 24 and 48 h after infection with pNL4-3 at 0.5 MOI and extracting DNA from overexpressed TZM-bl cells. Paired t test analysis revealed *p* values < 0.05. Error bar depicts SD between three independent experiment. [C] cDNA detection at different time point after overexpression of PSF inside the cell.

**Additional file 8: Figure S8.** Docked structure of HIV-1 Integrase with 27-mer *ds*DNA, (PDB ID. 1WKN). The 27 mer dsDNA (U5) was found to be present near DDE motif i.e. D64, D116 and E152 in catalytic core domain (CCD) of full length (aa 1-288) HIV-1 Integrase protein.

**Additional file 9.** MD Simulation Movie Clip.

**Additional file 10: Figure S9.** MD Trajectory Analysis of PSF-IN-dsDNA ternary complex (A) Number of H-bonds presence in MD trajectory; (B) RMSD plot of HIV IN and dsDNA during 100 ns MD trajectory.

**Additional file 11: Figures S10–S14.** Mass spectra of PSF peptides obtained through LC/MS/MS.

## Abbreviations

HIV-1: Human immunodeficiency virus-1
IN: Integrase
LTR: Long Terminal Repeat
KD: Knockdown
RRM: RNA recognition motif
FFT: Fast Fourier Transform
LRT: Late reverse transcription
ERT: Early reverse transcription
3′P: 3′processing
STA: strand transfer assay
MD: Molecular dynamics

## References

[1] Turlure F, Devroe E, Silver PA, Engelman A (2004). Human cell proteins and human immunodeficiency virus DNA integration. Front Biosci.

[2] Kalpana GV, Marmon S, Wang W, Crabtree GR, Goff SP (1994). Binding and stimulation of HIV-1 integrase by a human homolog of yeast transcription factor SNF5. Science.

[3] Miller MD, Bushman FD (1995). HIV integration. Inil for integration?. Curr Biol.

[4] Farnet CM, Bushman FD (1997). HIV-1 cDNA integration: requirement of HMG I(Y) protein for function of preintegration complexes in vitro. Cell.

[5] Chen H, Engelman A (1998). The barrier-to-autointegration protein is a host factor for HIV type 1 integration. Proc Natl Acad Sci USA.

[6] Cherepanov P, Maertens G, Proost P, Devreese B, Van Beeumen J, Engelborghs Y, De Clercq E, Debyser Z (2003). HIV-1 integrase forms stable tetramers and associates with LEDGF/p75 protein inhuman cells. J Biol Chem.

[7] Singh PK, Plumb MR, Ferris AL, Iben JR, Wu X, Fadel HJ, Luke BT, Esnault C, Poeschla EM, Hughes SH, Kvaratskhelia M, Levin HL (2015). LEDGF/p75 interacts with mRNA splicing factors and targets HIV-1 integration to highly spliced genes. Genes Dev.

[8] Gallay P, Hope T, Chin D, Trono D (1997). HIV-1 infection of nondividing cells through the recognition of integrase by the importin/karyopherin pathway. Proc Natl Acad Sci USA.

[9] Levin A, Armon-Omer A, Rosenbluh J, Melamed-Book N, Graessmann A, Waigmann E, Loyter A (2009). Inhibition of HIV-1 integrase nuclear import and replication by a peptide bearing integrase putative nuclear localization signal. Retrovirology.

[10] Armon-Omer A, Graessmann A, Loyter A (2004). A synthetic peptide bearing the HIV-1 integrase161–173 amino acid residues mediates active nuclear import and binding to importin alpha: characterization of a functional nuclear localization signal. J Mol Biol.

[11] Demeulemeester J, Blokken J, De Houwer S, Dirix L, Klaassen H, Marchand A, Chaltin P, Christ F, Debyser Z (2018). Inhibitors of the integrase-transportin-SR2 interaction block HIV nuclear import. Retrovirology.

[12] Houwer SD, Demeulemeester J, Thys W, Rocha S, Dirix L, Gijsbers R, Christ F, Debyser Z (2014). The HIV-1 integrase mutant R263A/K264A Is 2-fold defective for TRN-SR2 binding and viral nuclear import. J Biol Chem.

[13] Jayappa KD, Ao Z, Wang X, Mouland AJ, Shekhar S, Yang X, Yao X (2015). Human immunodeficiency virus type 1 employs the cellular dynein light chain 1protein for reverse transcription through interaction with its integrase protein. J Virol.

[14] Violot S, Hong SS, Rakotobe D, Petit C, Gay B, Moreau K, Billaud G, Priet S, Sire J, Schwartz O, Mouscadet J-F, Boulanger P (2003). The human polycomb group EED protein interacts with the integrase of human immunodeficiency virus type 1. J Virol.

[15] Mulder LCF, Lisa A, Chakrabarti M, Muesing A (2002). Interaction of HIV-1 integrase with DNA repair protein hRad18. J Biol Chem.

[16] Sean P, Krainer AR, Caputi M (2014). HIV-1 transcription is regulated by splicing factor SRSF1. Nucl Acids Res.

[17] Mandal D, Feng Z, Stoltzfus CM (2010). Excessive RNA splicing and inhibition of HIV-1 replication induced by modified U1 small nuclear RNAs. J Virol.

[18] Ranji A, Boris-Lawrie K (2010). RNA helicases: emerging roles in viral replication and the host innate response. RNA Biol.

[19] Peng R, Hawkins I, Link AJ, Patton JG (2006). The splicing factor PSF is part of a large complex that assembles in the absence of pre-mRNA and contains all five snRNPs. RNA Biol.

[20] Peng R, Dye BT, Perez I, Barnard DC, Thompson AB, Patton JG (2002). PSF and p54nrb bind a conserved stem in U5 snRNA. RNA.

[21] Shav-Tal Y, Zipori D (2002). PSF and p54(nrb)/NonO-multi-functional nuclear proteins. FEBS Lett.

[22] Emili A, Shales M, McCracken S, Xie W, Tucker PW, Kobayashi R, Blencowe BJ, Ingles CJ (2002). Splicing and transcription-associated proteins PSF and p54nrb/nonO bind to the RNA polymerase II CTD. RNA.

[23] Kula A, Gharu L, Marcello M (2013). HIV-1 pre-mRNA commitment to Rev mediated export through PSF and Matrin3. J Virol.

[24] Zhang WW, Zhang L-X, Busch RK, Farres J, Busch H (1993). Purification and characterization of a DNA-binding heterodimer of 52 and 100 kDa from HeLa cells. Biochem J.

[25] Alexandre TA, Bernard SL (2000). Human 100-kDa homologous DNA-pairing protein is the splicing factor PSF and promotes DNA strand invasion. Nucl Acids Res.

[26] Rajesh C, Baker DK, Pierce AK, Pittman DL (2011). The splicing-factor related protein SFPQ/PSF interacts with RAD51D and is necessary for homology-directed repair and sister chromatid cohesion. Nucl Acids Res.

[27] Zolotukhin AS, Michalowski D, Bear J, Smulevitch SV, Traish AM, Peng R, Patton J, Shatsky IN, Felber BK (2003). PSF acts through the human immunodeficiency virus type 1 mRNA instability elements to regulate virus expression. Mol Cell Biol.

[28] Straub T, Grue P (1998). The RNA-splicing factor PSF/p54 controls DNA-topoisomerase I activity by a direct interaction. J Biol Chem.

[29] Singh PK, Plumb MR (2015). LEDGF/p75 interacts with mRNA splicing factors and targets HIV-1 integration to highly spliced genes. Genes Dev.

[30] Tsukahara T, Matsuda Y, Haniu H (2013). PSF knockdown enhances apoptosis via downregulation of LC3B in human colon cancer cells. Biomed Res Int.

[31] Tandon V, Urvashi, Yadav P, Sur S, Abbat S, Tiwari V, Hewer R, Papathanasopoulos MA, Raja R, Banerjea AC, Verma AK, Kukreti S, Bharatam PV (2015). Design, synthesis, and biological evaluation of 12-dihydroisoquinolines as HIV-1 integrase inhibitors. ACS Med Chem Lett.

[32] Kessl JJ, McKee CJ, Eidahl JO, Shkriabai N, Katz A, Kvaratskhelia M (2009). HIV-1 integrase-DNA recognition mechanisms. Viruses.

[33] Métifiot M, Maddali K, Naumova A, Zhang X, Marchand C, Pommier Y (2010). Biochemical and pharmacological analyses of HIV-1 integrase flexible loop mutants resistant to raltegravir. Biochemistry.

[34] Krishnan L, Li X, Naraharisetty HL, Hare S, Cherepanov P, Engelman A (2010). Structure-based modeling of the functional HIV-1 intasome and its inhibition. Proc Natl Acad Sci USA.

[35] Cosnefroy O, Tocco A, Lesbats P, Thierry S, Calmels C, Wiktorowicz T, Reigadas S, Kwon Y, De Cian A, Desfarges S, Bonot P, San Filippo J, Litvak S, Cam EL, Rethwilm A, Fleury H, Connell PP, Sung P, Delelis O, Andréola ML, Parissi V (2012). Stimulation of the human RAD51 nucleofilament restricts HIV-1. Integration in vitro and in infected cells. J Virol.

[36] Nguyen TN, Goodrich JA (2006). Protein-protein interaction assays: eliminating false positive interactions. Nat Methods.

[37] Platt EJ, Bilska M, Kozak SL, Kabat D, Montefiori DC (2009). Evidence that ecotropic murine leukemia virus contamination in TZM-bl cells does not affect the outcome of neutralizing antibody assays with human immunodeficiency virus type 1. J Virol.

[38] Clouse KA, Powell D, Washington I, Poli G, Strebel K, Farrar W, Barstad P, Kovacs J, Fauci AS, Folks TM (1989). Monokine regulation of human immunodeficiency virus-1 expression in a chronically infected human T cell clone. J Immunol.

[39] Yoder KE, Fishel R (2008). Real-time quantitative PCR and fast QPCR have similar sensitivity and accuracy with HIV cDNA late reverse transcripts and 2-LTR circles. J Virol Methods.

[40] Anisenko AN, Knyazhanskaya ES, Zalevsky AO, Agapkina JY, Sizov AI, Zatsepin TS, Gottikh MB (2017). Characterization of HIV-1 integrase interaction with human Ku70 protein and initial implications for drug targeting. Sci Rep.

[41] Patton JG, Porto EB, Galceran J, Tempst P, Ginard BN (1993). Cloning and characterization of PSF, a novel pre-mRNA splicing factor. Genes Dev.

[42] Jenkins TM, Engelman A, Ghirlando R, Craigie RA (1996). Soluble active mutant of HIV-1 integrase: involvement of both the core and carboxyl-terminal domains in multimerization. J Biol Chem.

[43] Sherman PA, Fyfe JA (1990). Human immunodeficiency virus integration protein expressed in *Escherichia coli* possesses selective DNA cleaving activity. Proc Natl Acad Sci USA.

[44] Engelman A, Craigie R (1992). Identification of conserved amino acid residues critical for human immunodeficiency virus type 1 integrase function in vitro. J Virol.

[45] Li M, Craigie R (2005). Processing of viral DNA ends channels the HIV-1 integration reaction to concerted integration. J Biol Chem.

[46] Engelman A, Hickman AB, Craige R (1994). The core and carboxyl-terminal domains of the integrase protein of human immunodeficiency virus type 1 each contribute to nonspecific DNA binding. J Virol.

[47] Robinson WE, Reinecke MG, Abdel-Malek S, Jia Q, Chow SA (1996). Inhibitors of HIV-1 replication that inhibit HIV integrase. Proc Nat Acad Sci USA.

[48] Parissi V, Calmels C, De Soultrait VR, Caumont A, Fournier M, Chaignepain S, Litvak S (2001). Functional interactions of human immunodeficiency virus type 1 integrase with human and yeast HSP60. J Virol.

[49] Vandekerckhove L, Christ F, Maele BV, Rijck JD, Gijsbers R, Haute CV, Witvrouw M, Debyser Z (2006). Transient and stable knockdown of the integrase cofactor LEDGF/p75 reveals its role in the replication cycle of human immunodeficiency virus. J Virol.

[50] Lau A, Kanaar R, Jackson SP, O’Connor MJ (2004). Suppression of retroviral infection by the RAD52 DNA repair protein. EMBO J.

[51] Lloyd AG, Tateishi S, Bieniasz PD, Muesing MA, Yamaizumi M, Mulder LCF (2006). Effect of DNA repair protein Rad18 on viral infection. PLoS Pathog.

[52] Yoder K, Sarasin A, Kraemer K, McIlhatton M, Bushman F, Fishel R (2006). The DNA repair genes XPB and XPD defend cells from retroviral infection. Proc Natl Acad Sci USA.

[53] Montefiori DC (2004). Evaluating neutralizing antibodies against HIV, SIV and SHIV in luciferase reporter gene assays. Curr Proto Immunol.

[54] Li M, Gao F, Mascola JR, Stamatatos L, Polonis VR, Koutsoukos M, Voss G, Goepfert P, Gilbert P, Greene KM, Bilska M, Kothe DL, Salazar-Gonzalez JF, Wei X, Decker JM, Hahn BH, Montefiori DC (2005). Human immunodeficiency virus type 1 env clones from acute and early subtype B infections for standardized assessments of vaccine-elicited neutralizing antibodies. J Virol.

[55] Butler SL, Hansen MS, Bushman FD (2001). A quantitative assay for HIV DNA integration in vivo. Nat Med.

[56] Yun JJ, Heisler LE, Hwang IIL, Wilkins O, Lau SK, Hyrcza M, Jayabalasingham B, Jin J, McLaurin J, Tsao M-S, Der SD (2006). Genomic DNA functions as a universal external standard in quantitative real-time PCR. Nucl Acids Res.

[57] Maele VB, Rijck JD, Clercq ED, Debyser Z (2003). Impact of the central polypurine tract on the kinetics of human immunodeficiency virus type 1 vector transduction. J Virol.

[58] O’Doherty U, Swiggard WJ, Jeyakumar D, McGain D, Malim MH (2002). A sensitive, quantitative assay for human immunodeficiency virus type 1 integration. J Virol.

[59] Friedrich B, Li G, Dziuba N, Ferguson MR (2010). Quantitative PCR used to assess HIV-1 integration and 2-LTR circle formation in human macrophages, peripheral blood lymphocytes and a CD4 + cell line. Virol J.

[60] Liszewski MK, Yu JJ, O’Doherty U (2009). Detecting HIV-1 integration by repetitive-sampling Alu-gag PCR. Methods.

[61] Pierce BG, Wiehe K, Hwang H, Kim BH, Vreven T, Weng Z (2014). ZDOCK server: interactive docking prediction of protein–protein complexes and symmetric multimers. Bioinformatics.

[62] De Luca L, Pedretti A, Vistoli G, Barreca ML, Villa L, Monforte P, Chimirri A (2003). Analysis of the full-length integrase-DNA complex by a modified approach for DNA docking. Biochem Biophys Res Commun.

[63] Lee M, Sadowska A, Bekere I, Ho D, Gully BS, Lu Y, Iyer KS, Trewhella J, Fox AH, Bond CS (2015). The structure of human SFPQ reveals a coiled-coil mediated polymer essential for functional aggregation in gene regulation. Nucl Acids Res.

[64] Accelrys discovery studio visualizer, Version 4.5; software for visualizing and analyzing protein structures; Accelrys: San Diego, CA, 2017.

[65] Case DA, Cheatham TE, Darden T, Gohlke H, Luo R, Merz M, Onufriev A, Simmerling C, Wang B, Woods RJ (2005). The amber biomolecular simulation programs. J Comput Chem.

[66] Ryckaert JP, Ciccotti G, Berendsen HJC (1977). Numerical integration of the Cartesian equations of motion of a system with constraints: molecular dynamics of n-alkanes. J Comput Phys.

[67] Humphrey W, Dalke A, Schulten K (1996). VMD: visual molecular dynamics. J Mol Graph.

[68] Pettersen EF (2004). UCSF Chimera-a visualization system for exploratory research and analysis. J Comput Chem.

[69] Fogolari F, Brigo A, Molinari H (2003). Protocol for MM/PBS A molecular dynamics simulations of proteins. Biophys J.

[70] Miller BR (2012). MMPBSApy: an efficient program for end-state free energy calculations. J Chem Theory Comput.

