# Supplementary material for: Interaction of HIV-1 integrase with polypyrimidine tract binding protein and associated splicing factor (PSF) and its impact on HIV-1 replication
Source: Retrovirology. 2019 Apr 29;16:12. doi: 10.1186/s12977-019-0474-1 (PMC6489298; doi:10.1186/s12977-019-0474-1)

**Interaction of HIV-1 Integrase with Polypyrimidine tract binding protein and associated Splicing Factor (PSF) and its Impact on HIV-1 Replication**

Pooja Yadav^1^, Souvik Sur^2^, Dipen Desai^3^, Smita Kulkarni^3^, Vartika Sharma^4^, Vibha Tandon^1,2^

^1^Department of Chemistry, University of Delhi,Delhi-110007, India, ^2^Special Center for Molecular Medicine, JawaharLal Nehru University, New Delhi-110067, India. ^3^National AIDS Research Institute, Pune- 411026, Maharashtra, India. ^4^International centre for Genetics Enginering and Biotechnology, New Delhi-110067, India.

**Supplementary Information**

**Figure Legends**

**Additional file 1: Figure S1:** Histidine tag cleavage of IN using Thrombin.

**Additional file 2: Figure S2:** Measurement of cell viability percent in PSF knockdown and scrambled control cells by trypan blue exclusion dye.

**Additional file 3: Figure S3**: Transfection of GFP-PSF and GFP plasmid was performed in HEK 293T and TZM-bl cell line using lipofectamine 2000 and observed under fluorescence microscope (Olympus).

**Additional file 4: Figure S4**: The transfection efficiency was calculated after transfection with the 1 µg plasmid and percentage transfection was calculated by Fluorescence activated cell sorting (FACS). [A] Untransfected cells were shown as P3. [B] Transfection with GFP only with P4 region depicting the transfected cells. [C] Transfection with GFP-PSF with P4 showing the transfected cells.

**Additional file 5: Figure S5**: Cell viability was determined by 3-(4, 5Dimethylthiazol-2-yl)-2, 5 diphenyltetrazolium bromide) (MTT) reagent. TZM-bl cells were transfected with respective plasmid at concentration from 20 ng to 150 ng per well in triplicates and the percent viability or survival was determined by MTT after 24, 48 and 72 h.

**Additional file 6: Figure S6**: Analysis of overexpression of PSF on HIV replication as measured by luciferase reporter gene assay. [A] & [B] are the luciferase activity at 24 and 48 h at 0.1 and 0.5 MOI. TZM-bl cells were transfected with GFP-PSF plasmid. GFP with the same backbone was used as a control and viral replication was monitored. Data depicted here shows average values ±SD of 3 independent experiment. (**p* < 0.05).

**Additional file 7: Figure S7**: Quantitative PCR of HIV-1 2-LTR, integrated provirus and cDNA per cell - The TZM-bl cell line was overexpressed with the GFP-PSF plasmid and GFP was used as a control plasmid. Cells were transduced with the pNL4-3 virus at 0.5 MOI and DNA was detected by qPCR at different time points. [A] 2-LTR detection at different time point after overexpression of cell, infecting with pNL4-3 virus and harvesting DNA for analysis. [B] Integrated provirus was detected by Alu nested PCR at 24 and 48 h after infection with pNL4-3 at 0.5 MOI and extracting DNA from overexpressed TZM-bl cells. Paired t test analysis revealed *p* values < 0.05. Error bar depicts SD between three independent experiment. [C] cDNA detection at different time point after overexpression of PSF inside the cell.

**Additional file 8: Figure S8**: Docked structure of HIV-1 Integrase with 27-mer *ds*DNA, (PDB ID. 1WKN). The 27 mer dsDNA (U5) was found to be present near DDE motif i.e. D64, D116 and E152 in catalytic core domain (CCD) of full length (aa 1-288) HIV-1 Integrase protein.

**Additional file 9:** MD Simulation Movie Clip

**Additional file 10: Figure S9**: MD Trajectory Analysis of PSF-IN-dsDNA ternary complex (A) Number of H-bonds presence in MD trajectory; (B) RMSD plot of HIV IN and dsDNA during 100 ns MD trajectory.

**Additional file 11: Figure S10-S14:** Mass spectra of PSF peptides obtained through LC/MS/MS

**Additional file 6: Figure S6**


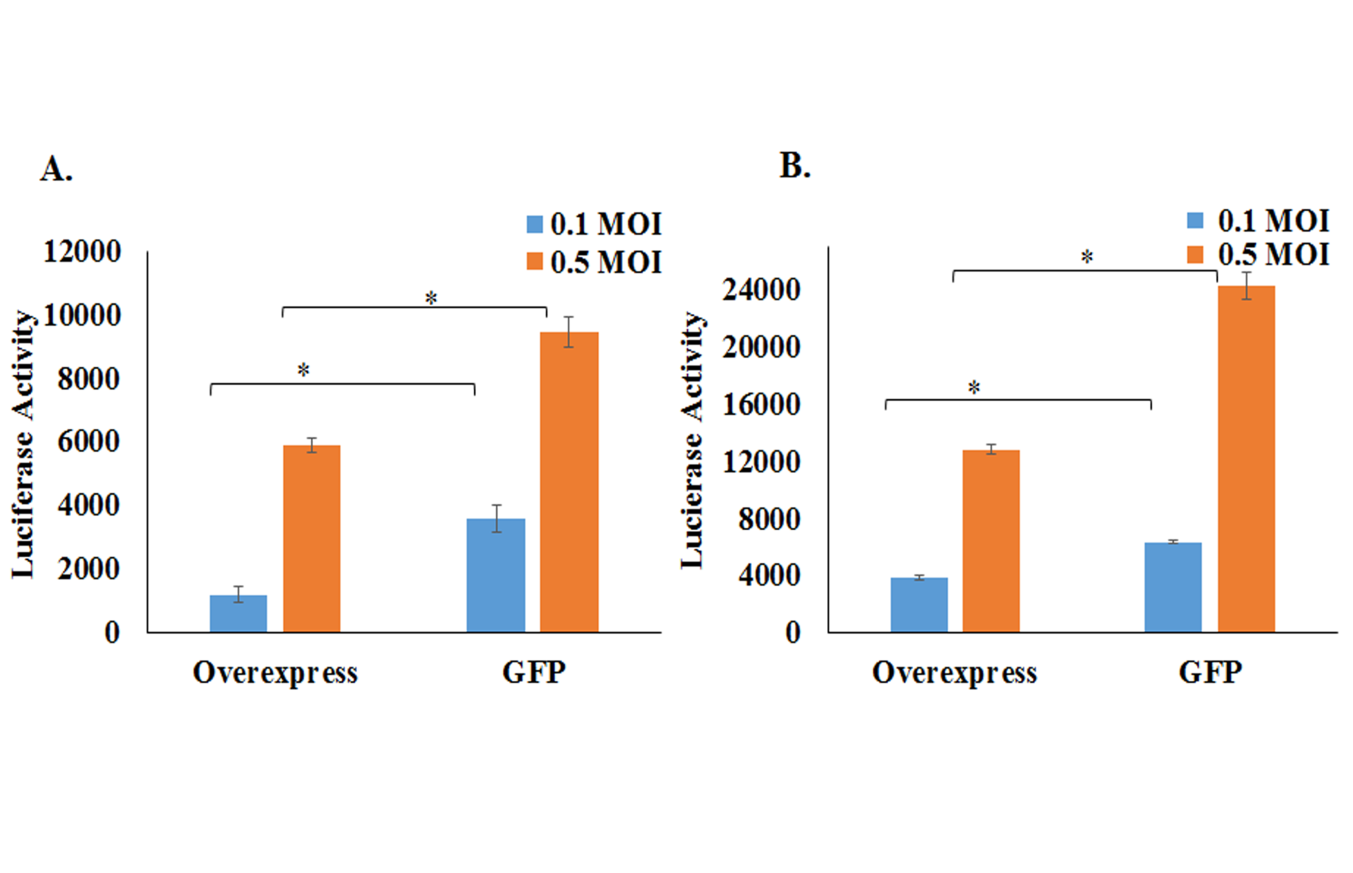

Supplement: Supplementary file 6 — Additional file 6: Figure S6. Analysis of overexpression of PSF on HIV replication as measured by luciferase reporter gene assay. [A] & [B] are the luciferase activity at 24 and 48 h at 0.1 and 0.5 MOI. TZM-bl cells were transfected with GFP-PSF plasmid. GFP with the same backbone was used as a control and viral replication was monitored. Data depicted here shows average values ± SD of 3 independent experiment. (*p < 0.05). [file 12977_2019_474_MOESM6_ESM.docx]
